# A growth‐ and bioluminescence‐based bioreporter for the *in vivo* detection of novel biocatalysts

**DOI:** 10.1111/1751-7915.12612

**Published:** 2017-04-10

**Authors:** Teunke van Rossum, Aleksandra Muras, Marco J.J. Baur, Sjoerd C.A. Creutzburg, John van der Oost, Servé W.M. Kengen

**Affiliations:** ^1^ Laboratory of MicrobiologyWageningen University and ResearchStippeneng 46708WE WageningenThe Netherlands

## Abstract

The use of bioreporters in high‐throughput screening for small molecules is generally laborious and/or expensive. The technology can be simplified by coupling the generation of a desired compound to cell survival, causing only positive cells to stay in the pool of generated variants. Here, a dual selection/screening system was developed for the *in vivo* detection of novel biocatalysts. The sensor part of the system is based on the transcriptional regulator AraC, which controls expression of both a selection reporter (LeuB or KmR; enabling growth) for rapid reduction of the initially large library size and a screening reporter (LuxCDABE; causing bioluminescence) for further quantification of the positive variants. Of four developed systems, the best system was the medium copy system with KmR as selection reporter. As a proof of principle, the system was tested for the selection of cells expressing an l‐arabinose isomerase derived from mesophilic *Escherichia coli* or thermophilic *Geobacillus thermodenitrificans*. A more than a millionfold enrichment of cells with l‐arabinose isomerase activity was demonstrated by selection and exclusion of false positives by screening. This dual selection/screening system is an important step towards an improved detection method for small molecules, and thereby for finding novel biocatalysts.

## Introduction

Research aiming at the development of whole‐cell bioreporters for a wide range of applications has increased substantially over the last few decades. Applications include detection of pollutants (Reed *et al*., 2012; Cho *et al*., 2014; Webster *et al*., 2014), the search for novel biocatalysts (Choi *et al*., 2014; Jha *et al*., 2014; Siedler *et al*., 2014a) and the improvement of strains for the industrial production of small molecules (Mahr and Frunzke, 2013; Schendzielorz *et al*., 2014; Siedler *et al*., 2014b). A whole‐cell bioreporter (hereafter referred to as bioreporter) is a living microorganism containing a sensor molecule that upon binding of a small molecule of interest switches on a reporter, resulting in a detectable phenotype (Hynninen and Virta, 2010; van der Meer and Belkin, 2010; Merulla *et al*., 2013). The high specificity of the sensor towards this small molecule together with the option to choose the reporter and thereby the way of measuring makes this method attractive. The potential to use bioreporters for high‐throughput screening explains the increased interest in these systems (Jeong *et al*., 2012; Ganesh *et al*., 2013; Mahr and Frunzke, 2013; Schallmey *et al*., 2014). For instance, various mutagenesis techniques lead to large numbers of altered production strains, but without a high‐throughput screening method, only a limited number of variants can be analysed (Binder *et al*., 2012; Schallmey *et al*., 2014; Schendzielorz *et al*., 2014). In the search for novel biocatalysts, screening large metagenomic or biocatalyst mutant libraries can be complicated and time‐consuming without a high‐throughput screening method, although in this field smart and focused libraries are emerging as well (Goldsmith and Tawfik, 2012; Illanes *et al*., 2012). Also other advantages have led to an increase in the use of bioreporters. These include high specificity, high enantioselectivity, lower costs, reduced handling, measuring bioavailability instead of actual concentration, no requirement of artificial substrates and the possibilities of online monitoring and signal enhancement (van der Meer *et al*., 2004; Gupta *et al*., 2012; Mahr and Frunzke, 2013; van Rossum *et al*., 2013).

The sensor part of the bioreporter can either function on transcriptional, translational or post‐translational level. Examples of sensors on the first two levels are transcriptional regulators and riboswitches/ribozymes respectively. On post‐translational level, various set‐ups are possible, for example a FRET (Förster resonance energy transfer) sensor, or a sensor directly coupled to enzyme activity (Michener *et al*., 2012). The specificity of the sensor towards the target molecule is essential in the functioning of the bioreporter. Obtaining the proper specificity can be time‐consuming. One can exploit nature, but for many small molecules no sensor is known yet (Hynninen and Virta, 2010; Gupta *et al*., 2012) and if there is one known, it cannot always be expressed heterologously (Jha *et al*., 2014). Another option is to engineer the specificity of a sensor, which may, however, demand a lot of time (Michener *et al*., 2012; van Rossum *et al*., 2013; Siedler *et al*., 2014b). Moreover, problems may arise, like the loss of protein stability (Schreier *et al*., 2009), or difficulties translating *in vitro* to *in vivo* if the initial screening is performed *in vitro* (Michener *et al*., 2012). Despite these hurdles, but due to their interesting properties, bioreporters are a growing practice and a lot of bioreporter‐related research is going on (Checa *et al*., 2012; Gredell *et al*., 2012; Michener *et al*., 2012; Park *et al*., 2013; Schallmey *et al*., 2014).

The reporter part of the bioreporter gives the cell a distinguishable phenotype, such as fluorescence, bioluminescence, colour, conditional survival, acidification of the environment or cell motility. Which type of reporter is used mainly depends on the available equipment and the desired characteristics such as dynamic range and sensitivity. Reporters that are most often used are green fluorescent protein (GFP), bacterial luciferase (LuxAB or LuxCDABE) and β‐galactosidase (LacZ). All three reporters are screening reporters, meaning that all cells, both negative and positive, stay in the pool (Boersma *et al*., 2007). Also with all three methods, the concentration of the molecule of interest can be quantified. However, high‐throughput screening with these reporters is often still laborious or expensive because of the requirement of microtiter plate assays or of fluorescence‐activated cell sorting (FACS) respectively. A simple, high‐throughput alternative is the use of a selection reporter instead of a screening reporter, which, by providing cell survival, causes only positive variants to stay in the pool. Although selection based on growth is rather straightforward and cheap, these are not yet broadly applied (van Sint Fiet *et al*., 2006; Choi *et al*., 2014).

The aim of this study was to develop a selection‐based reporter system for the detection of small molecules or more particularly for products of novel biocatalysts, and characterize its behaviour with respect to leakiness, maximal signal, dynamic range and sensitivity. More specifically, the developed system makes use of double reporters, consisting of both a selection reporter and a screening reporter, which allow for a rapid reduction of the initially large library size based on growth as well as subsequent quantification of the positive hits. Detection is based on the binding of the product of an enzyme reaction to a transcriptional regulator, resulting in a conformational change that alters its DNA‐binding capacity. This allows expression of the two divergently transcribed reporter genes. The selection reporter enables growth of the *Escherichia coli* cell, meaning that only cells in which the enzyme product is present, and thus express the active enzyme, will survive. The survivors can subsequently be screened using the screening reporter.

Here, different versions of the developed selection and screening system, varying in plasmid copy number and selection reporter, were compared in induction assays. The best performing system was the medium copy system with KmR as selection reporter. This system was used to detect the l‐arabinose isomerases derived from mesophilic *Escherichia coli* and thermophilic *Geobacillus thermodenitrificans* with l‐ribulose as substrate. Moreover, making use of the selection reporter, cells with one of the two l‐arabinose isomerases were enriched over cells without l‐arabinose isomerase. The screening reporter enabled the distinction of true from false positives.

## Results and discussion

### Components of the system

**Figure 1:**
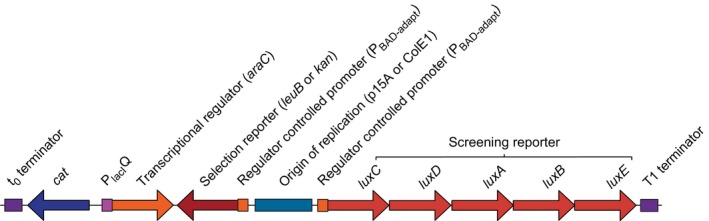
Linear representation of the regulator–reporter plasmid. Different versions of the plasmid vary in the selection reporter (*leuB* or *kan*) and the copy number of the regulator–reporter plasmid (ColE1 or p15A origins of replications for medium or low copy number respectively). The t0 terminator blocks readthrough transcription coming from the selection reporter or the chloramphenicol resistance marker (*cat*), whereas the T1 terminator blocks readthrough transcription from the screening reporter *luxCDABE*. P_lac_ _I_^Q^ is a moderate constitutive promoter. P_BAD__‐adapt_ is regulated by AraC.

To develop a sensitive double‐reporter system, with a broad dynamic range, high sensitivity and no leakage, four different versions were constructed and their performance was compared. To simplify the comparison, a plasmid‐based system was chosen, but for future work chromosomal integration might be preferred, to enhance stability and to reduce the use of antibiotics. Each system consisted of a host strain (*E. coli* BW25113 derivatives) and a regulator–reporter plasmid, encoding the transcriptional regulator and both reporters (Fig. 1). The two reporters were divergently transcribed to prevent readthrough transcription from one to the other. In the different system versions, the selection reporter and the plasmid copy number were varied.

As transcriptional regulator, we selected AraC, because it has been extensively studied and a protein structure is available with and without ligand. In particular, the last criterion is important in further studies in which we want to design variants in which the binding specificity of the regulator towards a small molecule of interest is adjusted. Also, this regulator has previously been engineered to alter its ligand specificity (Firestine *et al*., 2000; Tang *et al*., 2008, 2013; Tang and Cirino, 2011). In short, AraC is a dimer of which each monomer binds to one of two distant operator half sites upstream the *araBAD* operon, repressing its expression. Upon binding of l‐arabinose to AraC, DNA‐binding domains are reoriented to bind two more closely located half sites, allowing the *araBAD* operon to be transcribed and l‐arabinose to be metabolized. AraC also regulates its own gene, a gene of unknown function (*araJ*), genes involved in l‐arabinose transport (*araFGH* and *araE)* and several genes that are not directly implicated in arabinose metabolism (Schleif, 2010; Stringer *et al*., 2014). The arabinose regulon is also activated by the global regulator CRP (cAMP receptor protein) in response to low glucose levels (Kolb *et al*., 1993; Schleif, 2010). In this study, the natural inducer l‐arabinose was used for AraC and the pBAD promoter had a randomized CRP recognition site to make sure that reporter transcription was only regulated by AraC.

For selection, two different strategies for cell survival were compared, namely antibiotic resistance (kanamycin, KmR) and auxotrophy complementation (leucine, LeuB). Kanamycin resistance is realized by the aminoglycoside 3′‐phosphotransferase that impairs kanamycin binding to the 30S ribosomal subunit by adding a phosphate group to this aminoglycoside (Wright and Thompson, 1999). LeuB is a 3‐isopropylmalate dehydrogenase and is essential for l‐leucine biosynthesis (Somers *et al*., 1973). Only when this protein is present, cells can survive in the absence of l‐leucine. As the plasmid copy number may affect the behaviour of the reporter system, we constructed low and medium copy systems, by introducing the replication origins p15A and ColE1 respectively. For screening, bioluminescence was chosen, because it is very sensitive, has a broad dynamic range and is quickly detectable after induction. Moreover, no substrate is required when the whole operon *luxCDABE* is present (except FMNH_2_ and O_2_). The screening reporter genes used were in all systems *luxCDABE* from *Photorhabdus luminescens*, encoding the luciferase LuxAB and the multienzyme complex LuxCDE (LuxC, reductase; LuxD, transferase; LuxE, synthetase) that converts myristoyl‐acyl‐carrier protein to myristyl aldehyde, the substrate for the luciferase (Hakkila *et al*., 2002; Close *et al*., 2009; Park *et al*., 2013).

### Construction of the system

The construction of the system involved a series of cloning steps (Fig. S1) to make the regulator–reporter and control plasmids (Fig. 1), and the formation of several knockout strains. Each system module in the plasmids is flanked by unique restriction sites, allowing individual replacements. For each of the four regulator–reporter plasmids, two control plasmids were constructed, one for selection and one for screening. The ideal control would be an active site mutant of the reporter, because it is most similar to the actual system in terms of plasmid size, copy number, transcriptional and translational burden and therefore growth rate. However, as these reporter mutants were unavailable, an alternative approach was chosen here. A frameshift was made, either in the selection reporter gene (*kan*/*leuB*) or in one of the screening reporter genes (*luxA*). Compared with, for example, removal of the coding sequence (CDS), these controls are very similar to the parent plasmids regarding plasmid size and transcriptional and translational burden. The obtained sequences for the frameshift in the *kan* and the *leuB* genes differed from the expected fill in and removal of 5′ and 3′ overhangs respectively. Details and explanations are given in Table S1.

*Escherichia coli* BW25113 (Datsenko and Wanner, 2000) was used as host strain for the regulator–reporter plasmids and the control plasmids. This strain has a deletion in the *araBAD* operon (Grenier *et al*., 2014). It is therefore unable to metabolize arabinose (Morgan‐Kiss *et al*., 2002). Here, the genes *araC*,* leuB* and *recA* were deleted to exclude interference of endogenous AraC, to enable leucine auxotrophy complementation with LeuB and to prevent recombination events involving the plasmids respectively. Genes were replaced by a kanamycin resistance marker, which was later removed. Initially, the marker was removed by recombination of the flanking FLP recognition target (FRT) sites by FLP recombinase (Datsenko and Wanner, 2000). However, in subsequent gene deletions, the scar FRT site is still recognizable by FLP and hence not suitable. Therefore, the marker was flanked with *lox71*/*lox66* sites instead, of which the scar after recombination by Cre recombinase is no longer recognizable by Cre (Albert *et al*., 1995). The two obtained knockout strains Δ*araC* Δ*recA* and Δ*araC* Δ*leuB* Δ*recA* are indicated by AR and ALR in the rest of the text respectively.

After transformation of the knockout strains with the regulator–reporter or control plasmids, the relative copy numbers were determined. The relative plasmid copy number of the low and medium copy systems was 4–5 (Table S2). This ratio is slightly higher than copy number ratios reported for the pZ expression vectors, the parent plasmids of pFU98 from which the regulator–reporter plasmids and control plasmids were derived. pZ vectors with p15A or ColE1 replication origins had copy numbers of 20–30 and 50–70 respectively (Lutz and Bujard, 1997). However, as this study's plasmids are larger and have some different genes encoded, their demand on the cellular machinery and the building blocks might deviate, thereby altering the plasmid copy number. In addition, the pZ copy numbers were determined by comparing the activity of the plasmid‐encoded with the chromosome‐encoded luciferase (single copy). The ratio between frameshift control and parent plasmid was 1.0, confirming the expected similarity between the controls and their parent plasmids.

### Characterization of the selection reporter LeuB

All systems were characterized to determine their performance in selection and screening. In this context, a good performance means a low leakiness, a high maximal signal, a broad dynamic range and a high sensitivity. In the selection step of this system, a high sensitivity and low leakiness are the most important criteria to detect even low concentrations of the small molecule of interest without many false positives. Every cell that survives is interesting and the reporter signal will subsequently be quantified in the screening step, in which all four performance criteria are of importance, especially a high sensitivity and a broad dynamic range to obtain a relative ranking. In induction assays, the systems were induced by various concentrations of l‐arabinose. LeuB‐based assays were performed in minimal M9 medium, whereas KmR‐ and LuxCDABE‐based assays were performed in rich LB medium. The reporter activity or output was quantified by measuring the optical density (OD600) and/or the bioluminescence. This paragraph describes the results of the selection assay based on leucine auxotrophy complementation by LeuB.

**Figure 2:**
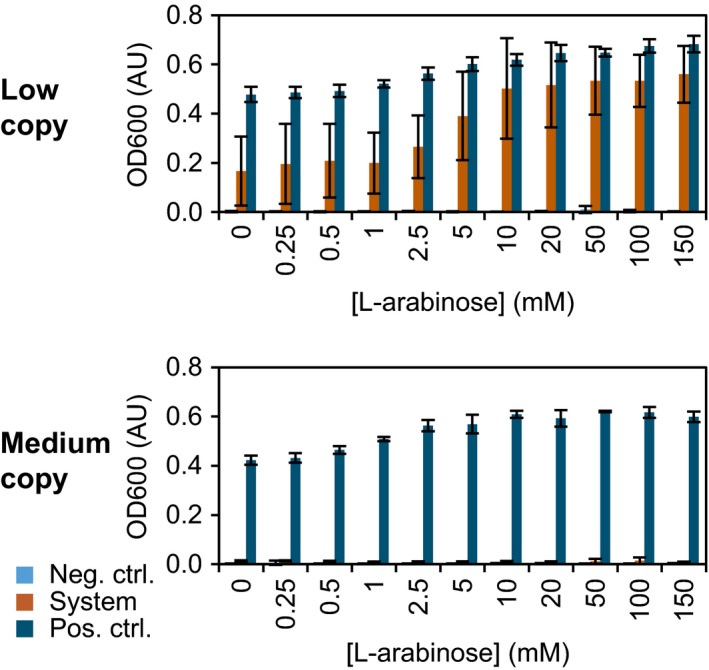
Selection based on leucine auxotrophy complementation. The plasmid‐encoded reporter gene *leuB* was induced in low and medium copy systems by various concentrations of the inducer l‐arabinose. Bacteria were grown in M9 medium for 32 h. The data are an average of three independent experiments (standard deviation indicated). System: auxotroph *E. coli* BW25113 Δ*araC* Δ*leuB* Δ*recA* (ALR) with the regulator–reporter plasmid. Neg. ctrl.: auxotroph ALR with the regulator–reporter plasmid with a frameshift in *leuB*. Pos. ctrl.: non‐auxotroph *E. coli* BW25113 Δ*araC* Δ*recA* (AR) with the regulator–reporter plasmid with a frameshift in *leuB*.

In the leucine auxotrophy complementation assay, the low and medium copy versions were analysed (Fig. 2). Three strains were tested for each system: (i) the system itself (auxotroph ALR + regulator–reporter plasmid), (ii) a negative control (auxotroph ALR + regulator–reporter plasmid with a frameshift in *leuB*) and (iii) a positive control (non‐auxotroph AR + regulator–reporter plasmid with a frameshift in *leuB*). The strains were not induced in the precultures because pre‐induction did not influence survival in the assay (Fig. S2). Bacteria were grown for 32 h (Fig. 2) and 48 h (Fig. S3) in minimal M9 medium. After 32 h, the positive controls were in stationary phase (except at low l‐arabinose concentrations), whereas most system strains were not (except for the low copy system at high l‐arabinose concentrations). The higher the l‐arabinose concentration, the faster system strains reached stationary phase. In addition, the low copy system grew faster than the medium copy system. The medium copy system did only barely grow after 48 h and in an unstable manner (large standard deviations and no definite relation between inducer concentration and growth). It could be that in minimal medium without leucine, the burden of the medium copy system was too high for the auxotrophic cells. As growth of the positive controls was not much influenced by the copy number, it was the combination of the higher copy number and the dependence on the plasmid encoded LeuB that caused the troubled complementation in the medium copy system. Growth was somehow positively affected by higher l‐arabinose concentrations (see positive controls), but growth on l‐arabinose seemed unlikely as *E. coli* BW25113 does not have the *araBAD* operon. The increase in growth of the low copy system with higher l‐arabinose concentrations was larger than for the positive control, as the increase was due to both the induction of *leuB* and the positive growth effect of l‐arabinose. Under non‐selective conditions, the frameshift‐based controls indeed grew very similar to the system itself. Moreover, under selective conditions, their reporter activity, measured as growth, was negligible. The frameshift approach is therefore a good method to make controls and may also be used in other studies.

### Characterization of the selection reporter KmR

**Figure 3:**
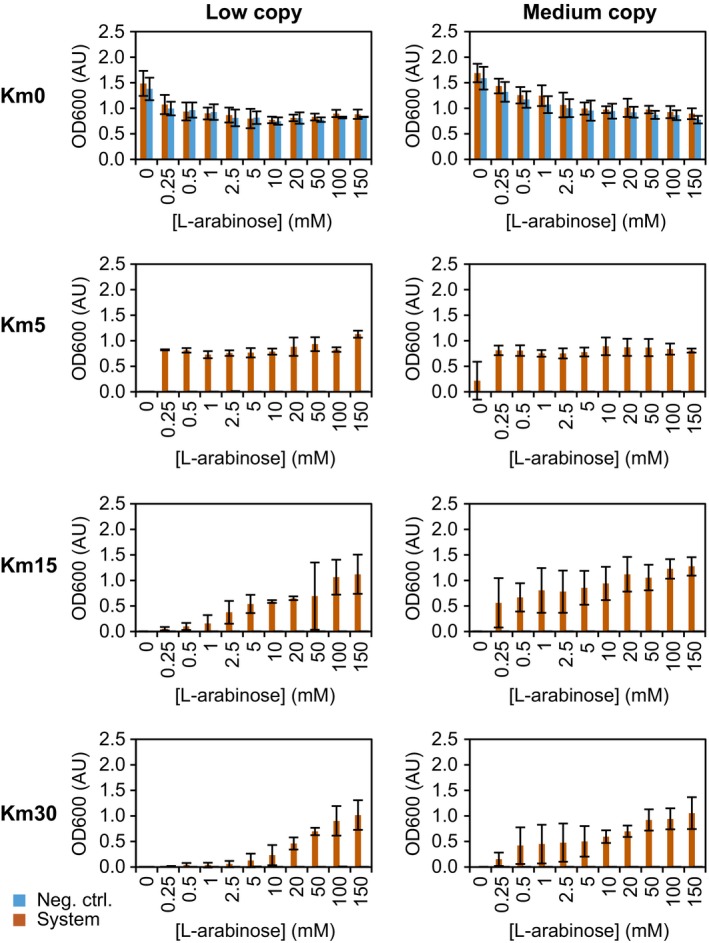
Selection based on kanamycin resistance. The plasmid‐encoded reporter gene *kan* was induced in the low and medium copy systems with the inducer l‐arabinose. Bacteria were grown in LB medium for 17 h in the presence of 0, 5, 15 or 30 μg ml^−1^ of kanamycin. The data are an average of three independent experiments (standard deviation indicated). System: *E. coli* BW25113 Δ*araC* Δ*recA* (AR) with the regulator–reporter plasmid. Neg. ctrl.: AR with the regulator–reporter plasmid with a frameshift in *kan*.

In the kanamycin resistance assay, the low and medium copy versions were analysed (Fig. 3). Two strains were tested for each system: (i) the system itself (AR + regulator–reporter plasmid) and (ii) a negative control (AR + regulator–reporter plasmid with a frameshift in *kan*). The strains were induced in the precultures (only non‐induced strains in the assays came from non‐induced precultures), because pre‐induction did affect survival in the assay (Fig. S4). The explanation of the pre‐induction effect was that l‐arabinose induces also expression of *araE*, encoding the low‐affinity l‐arabinose transport system. This inducer‐dependent transport control results in an all‐or‐nothing induction, in which intermediate l‐arabinose concentrations give rise to subpopulations of cells that are fully induced or non‐induced. The ratio of these subpopulations shifts over time towards full induction of all cells (Khlebnikov *et al*., 2000). This stage is most likely reached in the precultures, explaining the positive effect of pre‐induction on growth in the assay.

Bacteria were grown for 17 h in LB medium (stationary phase) in the presence of 0, 5, 15 and 30 μg ml^−1^ of kanamycin. These concentrations were chosen based on death curves at a fixed inducer concentration (Fig. S5). The negative controls and non‐induced system strains could not survive above 2.5 μg ml^−1^ of kanamycin, a concentration comparable to literature [1–3 μg ml^−1^ of kanamycin (Kumar and Venkatesh, 2010)]. Induction by l‐arabinose enabled the system strains to survive above 2.5 μg ml^−1^ and higher inducer concentrations allowed survival at higher kanamycin concentrations. However, at maximum induction none of the strains could cope with 50 μg ml^−1^ of kanamycin, the concentration commonly used to maintain plasmids with the same kanamycin marker. As in this study the plasmids were large and contained eight genes, the expression per gene was probably relatively low and not enough resistance was built up to deal with 50 μg ml^−1^ of kanamycin. Consistent with this, the lower copy system needed higher inducer concentrations than the medium copy system to deal with the same kanamycin concentration. This phenomenon of more gene copies, more protein and thus more resistance is called the gene dosage effect (Uhlin and Nordström, 1977). The relative low range of kanamycin concentrations should not be a problem, as long as future selections are performed within or just around this range. In contrast to the LeuB*‐*based assay, increasing the l‐arabinose concentration affected growth negatively (see 0 μg ml^−1^ of kanamycin). The opposite effect in the two assay types might be caused by the difference in growth medium, rich versus minimal medium. Unfortunately, a more detailed explanation cannot be given. Under non‐selective conditions, the frameshift‐based controls once more grew very similar to the system itself, and also here under selective conditions, their reporter activity, measured as growth, was negligible.

### Characterization of the screening reporter LuxCDABE

**Figure 4:**
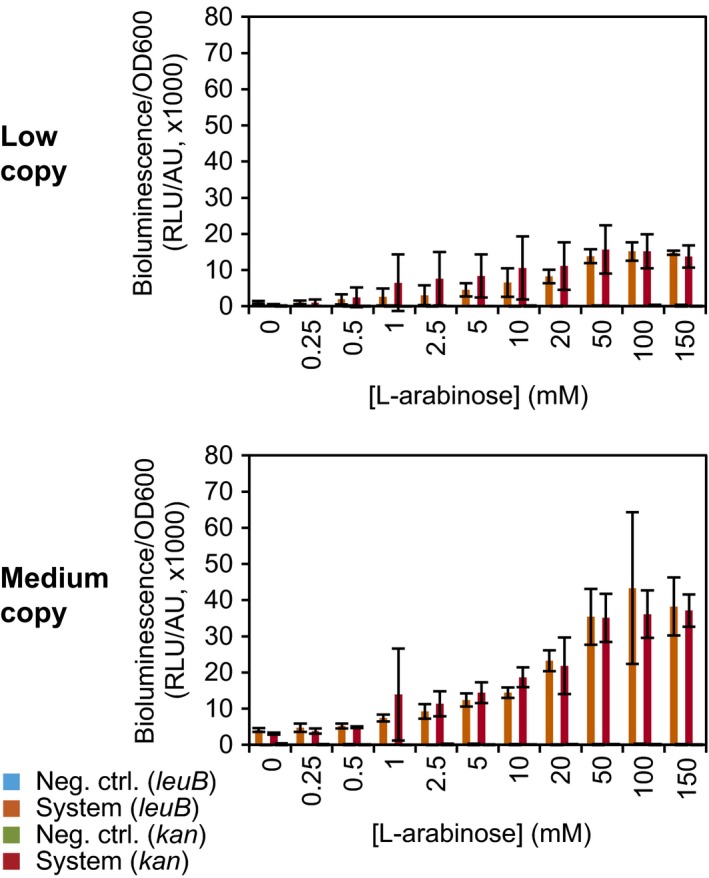
Screening based on bioluminescence. The plasmid‐encoded reporter operon *luxCDABE* was induced in four different systems by various concentrations of the inducer l‐arabinose. The four systems were the low and medium copy systems with either LeuB or KmR as selection reporter. Bacteria were grown in LB medium under non‐selective conditions for 5.5 h. The data are an average of three independent experiments (standard deviation indicated). System with LeuB: *E. coli* BW25113 Δ*araC* Δ*leuB* Δ*recA* (ALR) with the regulator–reporter plasmid with *leuB*. Neg. ctrl. with LeuB: ALR with the regulator–reporter plasmid with *leuB* and a frameshift in *luxA*. System with KmR: *E. coli* BW25113 Δ*araC* Δ*recA* (AR) with the regulator–reporter plasmid with *kan*. Neg. ctrl. with KmR: AR with the regulator–reporter plasmid with *kan* and a frameshift in *luxA*.

In the bioluminescence assay, all four systems were analysed (Fig. 4). Two strains were tested for each system: (i) the system itself (AR + regulator–reporter plasmid) and (ii) a negative control (AR + regulator–reporter plasmid with a frameshift in *luxA*). Bacteria were grown in LB medium for 5.5 h. At this time point, cultures were in late log phase at a point for which signal production and wash out due to cell division were about equal. Higher inducer concentrations resulted in more bioluminescence with maximal induction at 50 mM. These concentrations were comparable with literature values, namely 0.1–30 mM (Beverin *et al*., 1971; Shetty *et al*., 1999; Tang *et al*., 2008). The maximal induction for medium copy systems was higher than for low copy systems, probably a gene dosage effect. The KmR and LeuB versions did not differ in signal. The frameshift‐based controls again grew very similar to the system itself, and also here their reporter activity, measured as bioluminescence, was negligible. Comparing these systems with previous and future systems based on bioluminescence values will be difficult, because the energy state of the cell influences the bioluminescence. Slight differences in the protocol can already change the output. However, for the comparison of the systems within one study, this is not an issue.

### Comparison of the systems

To further compare the four systems, leakiness, maximal signal, dynamic range and sensitivity were determined (Table 1). Based on these characteristics, a comparison was made for (i) low versus medium copy and (ii) LeuB versus KmR.

**Table 1:** Characteristics of the reporter systems.^a^

Reporter	Copy number	Leakiness (AU)^b^		Maximal signal (AU)^c^		Dynamic range (mM)^d^		Sensitivity (mM)^e^	
LeuB	Low	0.16 ± 0.14	−	0.56 ± 0.12	− +	2.5–10	−	1.0–2.5	−
	Medium	NA	NA	NA	NA	NA	NA	NA	NA
KmR (Km5)	Low	0.00 ± 0.00	+ +	1.13 ± 0.07	+ +	0.25–0.25	− −	< 0.25	− +
	Medium	0.22 ± 0.37	− −	0.80 ± 0.04	+	0.25–0.25	− −	< 0.25	− +
KmR (Km15)	Low	0.00 ± 0.00	+ +	1.12 ± 0.38	+ +	0.25–100	+	< 0.25	− +
	Medium	0.00 ± 0.00	+ +	1.27 ± 0.18	+ +	0.25–20	− +	< 0.25	− +
KmR (Km30)	Low	0.00 ± 0.00	+ +	1.01 ± 0.29	+ +	10–150	−	5–10	−
	Medium	0.00 ± 0.00	+ +	1.05 ± 0.31	+ +	0.25–50	− +	< 0.25	− +
LuxCDABE (*leuB*)	Low	1058 ± 414	+	14599 ± 599	+	5−50	−	2.5–5	−
	Medium	4087 ± 507	+	38168 ± 8032	+ +	1−50	−	0.5–1	−
LuxCDABE (*kan*)	Low	349 ± 203	+ +	13684 ± 3101	+	5−50	−	2.5–5	−
	Medium	2960 ± 385	+	37076 ± 4436	+ +	0.5−50	− +	0.25–0.5	−

**a.** The systems vary in the selection reporter (LeuB or KmR) and the copy number of the regulator–reporter plasmid (medium or low). The KmR‐based systems are characterized at three different kanamycin concentrations (5, 15 and 30 μg ml^−1^). The LuxCDABE‐based systems are characterized for both LeuB and KmR containing versions. The standard deviation is included. A qualitative ranking is made (− −, −, − +, +, + +) with − − indicating a poor system and + + a good system. For leakiness, this indication is relative to the maximal signal. Absolute numbers for leakiness and maximal signal cannot be directly compared between the selection reporters LeuB and KmR and the screening reporter LuxCDABE, because they represent growth and bioluminescence respectively.

**b.** Signal at 0 mM inducer.

**c.** Signal at saturating inducer concentration.

**d.** Range of concentrations giving a changeable signal.

**e.** Lowest detectable inducer concentration.

#### Low versus medium copy

In the LeuB‐based assay, the growth rate of the medium copy system was unstable compared with the low copy system (Fig. 2 and Fig. S3), making determination of the four characteristics impossible. The medium copy system did not function very well, possibly because the auxotrophic cells were more burdened by the higher copy number in combination with the relative harsh condition of minimal medium without leucine. In the KmR‐based assay in general, low and medium copy systems were both not leaky, they had a similar maximal signal, but the medium copy system was more sensitive than the low copy system and the relative dynamic range of the two systems depended on the kanamycin concentration. Most likely, there was some expression in the absence of inducer; only the amount of KmR was not enough to deal with the lowest tested kanamycin concentration of 5 μg ml^−1^, appearing as if there was no leakiness. In contrast to the low copy system, the medium copy version had sufficient *kan* expression to survive 5 μg ml^−1^ of kanamycin. This gene dosage effect is likewise observed in the leakiness in the LuxCDABE‐based assay and also the probable cause of the difference in sensitivity in the KmR‐based assay. The delicate balance of survival and death at 5 μg ml^−1^ of kanamycin promotes use of slightly higher kanamycin concentrations in future studies. In the LuxCDABE‐based assay, the low copy systems were less leaky, had a lower dynamic range and were less sensitive than the medium copy systems, due to an overall lower expression level (gene dosage effect).

#### LeuB versus KmR

LeuB‐based selection was leakier than KmR‐based selection, due to the threshold set by adding ≥ 5 μg ml^−1^ of kanamycin. In addition, LeuB‐based selection had a lower maximal signal, because growth in minimal medium compared with rich medium reduces the maximal OD600. The sensitivity and the dynamic range (latter only at higher kanamycin concentrations) were better with KmR than with LeuB. In the KmR‐based assay, the sensitivity could be varied by changing the kanamycin concentration, and the assay time is much less than for the LeuB‐based assay, due to a higher growth rate in rich medium. Both are interesting features for later applications. Remarkably, the ability to deal with the selection pressure was less than expected in both selection assays. For leucine auxotrophy complementation, the system strains grew much slower than the positive controls, and for kanamycin resistance, system strains could not deal with the commonly used 50 μg ml^−1^. The explanation is twofold. On the one hand, the plasmids are large and multiple genes have to be expressed, lowering the expression per gene. On the other hand, the CRP binding site is absent, preventing regulation of reporter expression by CRP and thus by glucose. Normally, the presence of both cAMP (low glucose) and l‐arabinose does result in a higher induction than with l‐arabinose alone (Lis and Schleif, 1973).

#### Overall

All systems were functional except for the medium copy system with LeuB as selection reporter. But which system functions best? Based on the different characteristics described above and the rationale that in selection, a high sensitivity and a low leakiness are the most important criteria, and in screening, a high sensitivity and a broad dynamic range, the medium copy system with KmR as selection reporter was selected as best system. Since in the selection step a high sensitivity and a low leakiness are the most important criteria to detect even low concentrations of the small molecule of interest without much false positives, the total lack of leakiness at higher kanamycin concentrations is very valuable in future applications. Everything that survives is interesting and will subsequently be quantified in the screening step, in which a high sensitivity and a broad dynamic range are the most important criteria. The bit of leakiness in screening with the best system is therefore not detrimental. For screening, the fold change of the maximal signal over the leakiness was about ten. This fold change is similar to those in other transcriptional regulator‐based systems (Mustafi *et al*., 2012; Jha *et al*., 2014), but it is higher than in riboswitch‐based systems (Desai and Gallivan, 2004). The sensitivity for both selection (< 0.25 mM) and screening (0.25–0.5 mM) is lower than the sensitivity of described screening‐based bioreporters that were applied in, for example, library screening or strain optimization (0.05–10 μM; (Gupta *et al*., 2012; Choi *et al*., 2013; Siedler *et al*., 2014b), but is still of biological relevance (see section on isomerase detection below). The dynamic range of the medium copy system with KmR was satisfactory for both selection and screening (two orders of magnitude) and is comparable to those in other transcriptional regulator‐based systems (Choi *et al*., 2013; Cho *et al*., 2014; Siedler *et al*., 2014b).

### Proof of principle for application in enzyme screening

The next step was to obtain a proof of principle that the best performing system would be suitable for enzyme screening. As target, the enzyme l‐arabinose isomerase or AraA was chosen, because this enzyme activity can be linked to the AraC‐based system. Moreover, this type of enzymes is interesting for industrial production of rare sugars, like the sweetener D‐tagatose, which is produced from D‐galactose as a side reaction of l‐arabinose isomerase (Xu *et al*., 2014). l‐arabinose isomerase catalyses the first reaction in l‐arabinose breakdown, namely the conversion of l‐arabinose to l‐ribulose (Englesberg, 1961). *E. coli* BW25113 (Datsenko and Wanner, 2000), the strain used to create the system, has a deletion in the *araBAD* operon (Grenier *et al*., 2014) and thus no endogenous l‐arabinose isomerase (AraA), l‐ribulosekinase (AraB) and l‐ribulose‐5‐phosphate 4‐epimerase (AraD). As the reaction equilibrium of the isomerase is in favour of l‐arabinose [l‐arabinose/l‐ribulose = 5–15 (Yamanaka, 1960; Tewari and Goldberg, 1985)] and the reaction is not pulled towards l‐ribulose without AraB, it is likely that l‐ribulose is converted to l‐arabinose under the growth conditions in this study. Uptake of l‐ribulose was expected, because *E. coli* MG1655, having an intact *araBAD* operon, could grow on l‐ribulose. To show the applicability of the system for enzyme discovery of different origin, the l‐arabinose isomerase from mesophilic *E. coli* and the predicted l‐arabinose isomerase from thermophilic *G. thermodenitrificans* T12 were chosen. The latter was annotated as l‐arabinose isomerase (60% and 93% amino acid identities with *E. coli* MG1655 AraA and *G. thermodenitrificans* CBG‐A1 AraA respectively), but its function was not yet experimentally verified. For constitutive expression of *araA*, a second low copy plasmid was used next to the medium copy KmR‐based reporter system.

**Figure 5:**
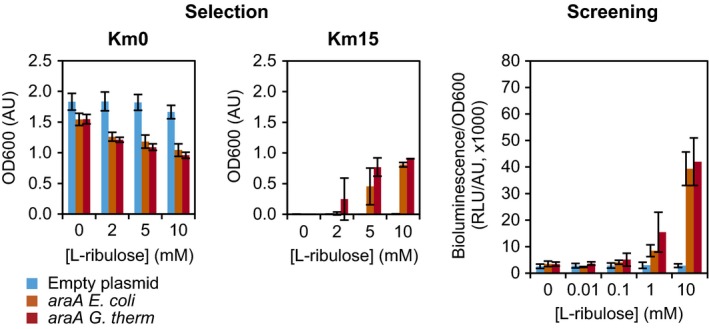
l‐arabinose isomerase detection by selection and screening assays. Conversion of l‐ribulose to l‐arabinose by the l‐arabinose isomerase AraA of *E. coli* or *G. thermodenitrificans* induced the system. Left (selection): detection based on kanamycin resistance. Bacteria were grown in LB medium for 17 h in the presence of 0 and 15 μg ml^−1^ of kanamycin. Right (screening): detection based on bioluminescence. Bacteria were grown in LB medium for 5.5 h. The data are an average of two or three independent experiments (standard deviation indicated) for selection or screening respectively. *araA E. coli* or *G. therm*:* E. coli* BW25113 Δ*araC* Δ*recA* (AR) with the regulator–reporter plasmid and the plasmid expressing *araA* of *E. coli* or *G. thermodenitrificans*. Empty plasmid: AR with the regulator–reporter plasmid and the empty plasmid.

To show that the system could indeed detect the activity of the two l‐arabinose isomerases, KmR‐ and LuxCDABE‐based assays were performed in which l‐ribulose was added to the medium as substrate for AraA (Fig. 5). The negative control was the system strain with the second plasmid lacking the *araA* CDS. For the KmR‐based assay, cells were grown in LB medium for 17 h with 0 or 15 μg ml^−1^ of kanamycin. Only when one of the l‐arabinose isomerases was expressed, cells survived the kanamycin, verifying the annotation of *G. thermodenitrificans* T12 *araA* and showing that the system is capable of detecting a mesophilic and a thermophilic enzyme based on growth. However, a substantial amount of l‐ribulose was needed to observe the enzyme activity, namely ~2 mM. This sensitivity differed an order of magnitude with the sensitivity for l‐arabinose of cells without l‐arabinose isomerase (~2 versus ~0.25 mM; Table 1). It was unlikely that this decrease in sensitivity was a result of a difference in uptake between the two sugars, because the sensitivity in the LuxCDABE‐based assay (see below) was in the same order of magnitude for extracellular added l‐arabinose or l‐ribulose converted to l‐arabinose. A more probable explanation was the burden of expressing *araA* (Fig. 5; Km0, empty plasmid versus *araA*). This burden had two components: the effect of *araA* on growth in the absence and in the presence of l‐ribulose. In the absence of l‐ribulose, cells expressing *araA* were hindered in growth (Fig. 5, stationary phase; Fig. S6, exponential phase). Whether it was the activity of AraA or just its expression load was not known, but the observation that *araA* was a burden to the cells was strengthened by the failure to make a plasmid with *E. coli araA* under the stronger P_lacUV5_ promoter. Cells expressing *E. coli araA* were more burdened than cells expressing *G. thermodenitrificans araA* (Fig. S6), possibly because they seemed to higher express *araA* (Fig. S7). Better expression of *E. coli araA* than *G. thermodenitrificans araA* was expected, because the latter was not expressed in its endogenous host. In the presence of l‐ribulose, cells were more burdened by *araA* than in the absence of l‐ribulose, and with higher l‐ribulose concentrations, the burden increased (Fig. 5). As mentioned above, l‐arabinose had a negative effect on growth and it is therefore most likely that the l‐arabinose formed out of l‐ribulose caused the concentration‐dependent growth defect. The system was slightly more sensitive for the *G. thermodenitrificans araA* than for the *E. coli araA* (< 2 versus 2–5 mM), probably due to the growth differences between the two strains. Cells with *G. thermodenitrificans araA* might have had a lower level of active *araA* due to a lower expression and a lower activity because of its thermophilic origin. Therefore, these cells had a less negative effect on growth from l‐arabinose compared with the cells with *E. coli araA*.

For the LuxCDABE‐based assay, cells were grown in LB medium for 5.5 h. Only when one of the l‐arabinose isomerases was expressed, cells were bioluminescent, showing that the system is also capable of detecting a mesophilic and a thermophilic enzyme based on bioluminescence. The sensitivity of this assay was similar for both l‐arabinose isomerases and about > 50‐fold higher than that of the KmR‐based assay (0.01–0.1 mM versus 2–5 mM). This difference was most likely caused by the negative growth effect of both l‐arabinose and AraA in the KmR‐ or growth‐based assay. As in this assay a threshold of expressed KmR had to be reached, a negative growth effect probably had a more detrimental effect than in the LuxCDABE‐based assay, having a more gradual response curve. Quantification of the different levels of enzyme activity was not as straightforward as envisioned due to the negative growth effect of l‐arabinose and the difference in expression levels between the *E. coli* and the *G. thermodenitrificans* l‐arabinose isomerase.

**Figure 6:**
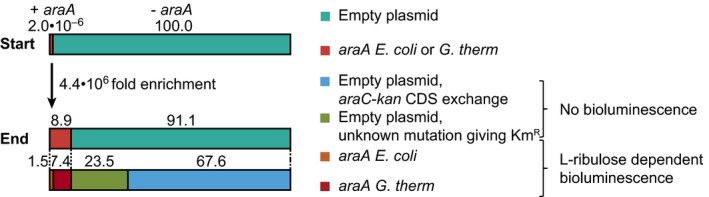
Enrichment of cells with l‐arabinose isomerase (AraA) activity. Cells with *E. coli araA, G. thermodenitrificans araA* or no *araA* were mixed in a 1:1:10^8^ ratio (2.0 × 10^−6^% of cells with *araA*) and cells with l‐arabinose isomerase activity were selected based on kanamycin resistance in the presence of 5 mM of l‐ribulose as substrate and 15 μg ml^−1^ of kanamycin. Selected colonies were analysed by a bioluminescence‐based screening assay in the presence of 0.5 mM of l‐ribulose to show the l‐ribulose‐dependent bioluminescence as verification of *araA* presence. Further verification was performed by PCR. *araA E. coli* or *G. therm*:* E. coli* BW25113 Δ*araC* Δ*recA* (AR) with the regulator–reporter plasmid and the plasmid expressing *araA* of *E. coli* or of *G. thermodenitrificans*. Empty plasmid, *araC*‐*kan* CDS exchange: AR with the regulator–reporter plasmid with a recombination of the *araC* and *kan* CDSs that places *kan* under a constitutive promoter instead of the AraC‐controlled promoter, and the empty plasmid. Empty plasmid, unknown mutation giving Km^R^: AR with the regulator–reporter plasmid and the empty plasmid and an unknown mutation giving kanamycin resistance. Values above bars are percentages.

Altogether, these assays showed that the system was capable of detecting a mesophilic and a thermophilic enzyme based on growth and on bioluminescence. However, to show that this system is suitable for application in enzyme screenings, it has to be able to enrich cells with the desired enzyme activity over cells that do not have this activity. For this purpose, selection and screening of an enzyme library was mimicked by mixing cells with the *E. coli araA, G. thermodenitrificans araA* or no *araA* (empty plasmid) in a 1:1:10^8^ ratio. Cells were selected based on kanamycin resistance for 6 h in liquid medium and 17 h on agar plates in the presence of 5 mM of l‐ribulose as substrate and 15 μg ml^−1^ of kanamycin. Making use of the second reporter, the 68 selected colonies were analysed by a bioluminescence‐based screening assay in the presence of 0.5 mM of l‐ribulose to show the l‐ribulose‐dependent bioluminescence as verification of *araA* presence. Six of these colonies gave l‐ribulose‐dependent bioluminescence (Fig. S8) and were verified by PCR to contain *araA*. The other colonies were false positives; they did not give bioluminescence and were verified by PCR to contain the empty plasmid. Based on the control cultures with only one strain, 25 times more *araA* containing cells were expected. The low number might have been caused by competition with false positives in the mixed culture. Of the six *araA* containing colonies, one colony had *araA* of *E. coli* and five colonies had *araA* of *G. thermodenitrificans* (Fig. 6). This advantage of the *G. thermodenitrificans araA* over the *E. coli araA* containing cells was due to their faster growth. During the 6 h in liquid medium, the cells with *G. thermodenitrificans araA* grew about six times faster than the cells with *E. coli araA* in the control cultures containing only one strain. They were less burdened by AraA and growth inhibiting l‐arabinose, as discussed above.

Starting from 2.0 × 10^−6^% of the cells having *araA* and ending with 8.8% (Fig. 6) meant an enrichment of 4.4 × 10^6^ fold in only one round of selection and screening. Other systems were just tested with initial ratios up to 1:10^6^ and required at least two FACS rounds or one selection round to get to a more than 10^5^ fold enrichment (van Sint Fiet *et al*., 2006; Copp *et al*., 2014; Jha *et al*., 2014). Thus, the system described here is able to obtain a very good enrichment, and it is relatively easy, short and cheap, compared with, for example, FACS. In addition, it is able to distinguish the false positives from true positives with the subsequent screening assay, emphasizing the value of this dual reporter system. Dietrich *et al. *already published a dual reporter system with TetA for selection and GFP for screening, but unfortunately the combined use of the two reporters was not yet fully demonstrated (Dietrich *et al*., 2013). Garmendia *et al. *successfully demonstrated another two stages approach, using *pyrF* as reporter gene in a Δ*pyrF* background. Positive selection was based on uracil auxotrophy complementation and negative selection based on fluoroorotic acid sensitivity (Galvão and de Lorenzo, 2005; Garmendia *et al*., 2008). The nature of the false positives was investigated by PCR and 74.2% (Fig. 6) of the false positives had a recombination in the regulator–reporter plasmid. A 17‐base pair region including the ribosomal binding site (RBS) in front of *kan* was recombined with the identical region in front of *araC*. This resulted in an exchange of the CDSs of these two genes, placing *kan* under the constitutive P_lacI_^Q^ instead of under the AraC‐controlled P_BAD‐adapt_ and thereby enabling the cells to survive kanamycin in the absence of *araA*. Interestingly, this recombination took place despite the deletion of *recA*. Although a fragment as short as 17 bp was not tested, *E. coli* is capable of RecA‐independent recombination of short homologous regions (Dutra *et al*., 2007). Adaptation of the system to prevent this recombination was not considered useful, because in that case other escape mutants are likely to become dominant as is intrinsic to selection. The nature of the false positives made the screening by bioluminescence as second step better than a second selection step in which the false positives would survive again. The other 25.8% of false positives had an unknown mutation giving constitutive resistance to 15 μg ml^−1^kanamycin. One possibility is a mutation in P_BAD‐adapt_ to make expression of *kan* independent of AraC.

## Conclusions

In this study, a selection‐based system for the detection of small molecules, or more particularly for products of novel biocatalysts, was developed and characterized. The system expresses two reporters under control of AraC, allowing for both selection (based on growth) and screening (based on bioluminescence). Growth‐based selection allows for a rapid reduction of the initially large library size and subsequent positive hits can be quantified by bioluminescence. Different versions of the system with a low or medium plasmid copy number and leucine auxotrophy complementation (LeuB) or kanamycin resistance (KmR) as selection reporter were compared. The medium copy system with KmR as selection reporter was selected as best system, based on leakiness, maximal signal, dynamic range and sensitivity in both selection and screening. This system was used to detect l‐arabinose isomerase derived from mesophilic *E. coli* and thermophilic *G. thermodenitrificans* with l‐ribulose as substrate. Moreover, cells with one of the two l‐arabinose isomerases were enriched over cells without l‐arabinose isomerase with a factor 4.4 × 10^6^, making use of the selection reporter. The screening reporter enabled the distinction of true from false positives.

Previous objections to bioreporters with growth‐based selection were that growth assays can have a relatively low dynamic range or low sensitivity, and a high level of false positives due to escape mutants, unanticipated survival mechanisms or various influences on growth of the positive cells (Taylor *et al*., 2001; Dietrich *et al*., 2013; van Rossum *et al*., 2013; Jha *et al*., 2014). In the systems described in this study, however, the dynamic range and sensitivity in selection were similar or even slightly better than in screening. Both dynamic range and sensitivity are comparable to other reported systems, but the sensitivity of the here reported system might need some improvement, e.g. via adaptation of the relative expression levels of the system components. Overall, the best performing system has an appropriate working range as confirmed by its ability to detect an enzyme activity as proof of principle. Moreover, the system is able to enrich cells with the enzyme activity over cells that do not have the activity on a scale mimicking a library of 10^8^, in a relatively easy, fast and cheap manner. The set‐up as double‐reporter system reduces the number of false positives by having the selection and screening steps in series, which function therefore as double‐check. Although the enrichment is already much better than for other systems, further improvements like an additional selection reporter under control of AraC or using a selection reporter that allows for both negative and positive selection could improve the selection potential and reduce the number of false positives even more. The modular make‐up of the system makes the exchange of components like the selection reporter straightforward. Also the screening reporter could be exchanged, for example by GFP, in cases where the dependency of the reporter activity on the metabolism or growth phase is a problem. Genome integration of the reporters might be an option to enhance the stability of the system. Noteworthy, each of these alterations requires some fine‐tuning and characterization.

Although a proof of principle for the application in enzyme searches is shown here, the system developed in this study should be regarded as a prototype. Application of this system in detecting specific small molecules requires changing the specificity of the system by altering the transcriptional regulator. Two approaches can be used to adjust the specificity. First, the system can be easily recloned to function with another transcriptional regulator, because the constructs have a modular design. In that case, the characteristics should be determined again, because they might differ due to distinct induction mechanisms or different transcriptional or translation rates of the regulators or dissimilar binding kinetics of the regulators to the DNA and to their inducers. Second, the transcriptional regulator can be engineered to change its inducer specificity as was carried out for AraC in other studies (Firestine *et al*., 2000; Tang *et al*., 2008, 2013; Tang and Cirino, 2011). Although less drastic changes in characteristics are envisioned than for a complete new regulator (promoter sequences, most of CDS, etc., stay the same), also in this case, characteristics should be determined again. A most interesting feature of the system is that the system itself can be used to select and optimize a new regulator variant. A library of transcriptional regulator variants can be made, and with the system, the variant with the highest specificity towards the target small molecule can be selected. Additional rounds of library formation and selection can further optimize the specificity. Although the double‐reporter system with its subsequent selection and screening steps reduces the number of false positives when detecting small molecules, a good counter selection is still required to reduce the number of false positives that originate from regulators that allow transcription of the reporter in the absence of the inducer. Also discrimination between variants that only differ slightly in specificity (Galvão and de Lorenzo, 2006) might require a more tight selection as described above. A combination of negative and positive selection, preferably accommodated by one gene, might proof useful.

In conclusion, this study provided insight into various aspects of whole‐cell bioreporters. The successful development is described of an alternative for the often expensive and/or laborious high‐throughput novel biocatalyst detection, and more general for small molecule detection, by combining a selection and a screening reporter in a single system. Future research will focus on the next crucial step, namely using the system for the selection of regulator variants.

## Experimental procedures

### Bacterial strains and media

*E. coli* DH10B T1^R^ (catalogue number C6400‐03; Invitrogen, Waltham, MA, USA) was used for plasmid propagation and was grown and transformed by standard methods (Sambrook *et al*., 1989). *E. coli* BW25113 JW0063‐1 of the KEIO collection (Baba *et al*., 2006) was the parent strain for the constructed knockout strains. The knockout strains hosted the regulator–reporter plasmids or their controls. Transformations were performed by electroporation (ECM 630 electroporator (BTX), 2500 V, 200 Ω, 25 μF, 2‐mm cuvettes, 20–50 μL of electrocompetent cells, recovery in LB medium). Cells were generally grown in LB medium with the appropriate antibiotics: 100 μg ml^−1^ of ampicillin, 50 μg ml^−1^ of kanamycin or 34 μg ml^−1^ of chloramphenicol, unless stated otherwise. Leucine auxotrophy complementation assays and growth on l‐ribulose were performed in M9 medium. Enrichments were performed in LB medium with 4 g l^−1^ of glycerol to reach a higher OD600.

### Construction of regulator–reporter plasmids and control plasmids

The regulator–reporter plasmids pWUR766 and pWUR768 (~10 kb each) were obtained in seven subsequent cloning steps from pFU98 (Uliczka *et al*., 2011; kindly provided by Petra Dersch). pFU98 contains a chloramphenicol resistance marker (*cat* encoding chloramphenicol acetyltransferase), the pSC101* origin of replication protected from readthrough transcription by two flanking terminators (t_0_ and T1), a multiple cloning site and a very strong RBS (AGGAGG; ‐12 to ‐7 relative to translation start) in front of *luxCDABE*. The cloning steps were (i) replacement of the very low copy origin pSC101* by the medium copy ColE1 to ease further cloning steps, (ii) insertion of the selection reporter gene *leuB* or *kan* (incl. RBS as above and PvuI site; for *leuB* silent mutation with same codon usage factor, TCG→AGT, to remove AatII and PvuI sites from CDS), (iii), insertion of the moderately strong and constitutive P_lacI_^Q^ promoter (Glascock and J. Weickert, 1998); incl. CpoI site), (iv) insertion of the transcriptional regulator gene *araC* (incl. RBS as above) behind P_lacI_^Q^, (v) insertion of the P_BAD‐adapt_ promoter and operator region in front of *luxCDABE*, (vi) translocation of ColE1 in between the two reporters to prevent expression and/or recombination problems by the two almost identical promoter sequences next to one another (the terminators were left at the original location) and (vii) insertion of P_BAD‐adapt_ in front of *leuB*/*kan*. P_BAD‐adapt_ (this study) had a randomized CRP binding site to make sure that the reporters are only regulated by AraC and it had an internal restriction site (NheI or PstI; Table S3). More details of the intermediary cloning steps and the primers are given in Fig. S1 and Table S4 respectively.

The origin ColE1 in pWUR766 and pWUR768 was replaced by p15A with Acc65I/AvrII to yield the low copy variants pWUR770 and pWUR772 respectively. From each of the four constructs, two control constructs were made containing a frameshift either in the selection reporter gene (*leuB* or *kan*) or in one gene of the screening reporter operon (*luxA*). The parent plasmids were digested inside the gene at a unique restriction site: Eam1105I in *leuB*, XagI in *kan* and Cfr42I in *luxA*. The ends were made blunt with Klenow fragment, according to the protocol of Thermo Scientific (Waltham, MA, USA).

For all cloning steps, plasmids were isolated with the Plasmid Miniprep kit of Thermo Scientific (#K0503). PCRs to create insert fragments were performed with Pfu. Vector fragments were treated with Antarctic Phosphatase (NEB, Ipswich, MA, USA), according to the protocol of NEB. Insert or vector fragments were purified with the PCR purification kit of Thermo Scientific (#K0702), the DNA Clean & Concentrator‐5 kit of Zymo Research (D4004; Irvine, CA, USA), or the gel extraction kits of Thermo Scientific (#K0692) or Zymo Research (D4002). Ligation was performed for 1 h at room temperature with T4 ligase. Cloning events were verified by PCR with DreamTaq and/or restriction analysis and by sequencing at GATC Biotech (Cologne, Germany). All enzymes were obtained from Thermo Scientific, unless stated otherwise. The nucleotide sequences of the four regulator–reporter plasmids pWUR766, pWUR768, pWUR770 and pWUR772 were submitted to the GenBank database under accession numbers KX670545‐8 respectively.

### Construction of knockout strains

The kanamycin resistance gene *kan* from *E. coli* BW25113 JW0063‐1 (Δ*araC*::*kan*) of the KEIO collection (Baba *et al*., 2006) was eliminated by FLP recombinase encoded on pCP20 (Cherepanov and Wackernagel, 1995) as described by Datsenko and Wanner (2000).

The Δ*araC* Δ*leuB* double knockout was constructed according to Datsenko and Wanner (2000); with the exception of the disruption cassette. A new disruption cassette was developed based on the recombination cassette from Westra *et al*. (2010); replacing the FRT sites that flank *kan* with *lox71*(left)/*lox66*(right) sites (Albert *et al*., 1995) synthesized and cloned SfiI/SfiI in pMA‐RQ by GeneArt AG (see Table S3 for description and sequence; Waltham, MA, USA). With this plasmid, pMA‐RQ_lox71_*kan*_lox66, as template, a linear cassette was created by PCR with Pfu (Thermo Scientific), introducing the homologous regions (same regions as in Baba *et al*., 2006). After direct DpnI treatment, the product was purified with the PCR purification kit of Thermo Scientific (#K0702). For elimination of *kan* by Cre recombinase, parts of the protocol from Datsenko and Wanner (2000); were replaced by components of the protocol from Palmeros *et al*. (2000). Knockouts were transformed with pJW168 (Wild *et al*., 1998), and transformants were selected on LB medium with ampicillin and 0.5 mM of IPTG at 30°C and cured from plasmids at 37°C. For the *leuB* deletion, leucine auxotrophy was verified on minimal M9 medium with or without 20 mg l^−1^ of l‐leucine. The parent strain was taken as control.

The Δ*araC* Δ*recA* double knockout and the Δ*araC* Δ*leuB* Δ*recA* triple knockout (designated AR and ALR respectively) were constructed as described above for Δ*araC* Δ*leuB* with one exception. The disruption cassette (same homologous regions as in Baba *et al*., 2006) was made with purified SfiI‐digested pMA‐RQ_lox71_*kan*_lox66 as template in the PCR, making DpnI treatment unnecessary. Elimination of *kan* was the same as for Δ*araC* Δ*leuB*.

Recombination events were verified by PCR with REDTaq (Sigma‐Aldrich, Steinheim, Germany) or DreamTaq (Thermo Scientific). Gene replacement by *kan* was verified with two primer sets, each set with one primer flanking the altered region and one inside *kan*. *kan* elimination was verified with one primer set, each primer flanking the altered region. All deletions in the two final knockout strains AR and ALR were verified by PCR with Pfu, and PCR products were sequenced at GATC Biotech. All primers are presented in Table S4. The knockout strains were transformed with the regulator–reporter plasmids or control plasmids.

### Induction assays

The three types of induction assays, based on expression of *leuB*,* kan* or *luxCDABE,* had a similar experimental set‐up. Two millilitres of precultures were inoculated from agar plates made of the same medium (for adaptation) and grown in 10 mL tubes (Gosselin, Hasebrouck, France). The assays were performed in 2 ml 96‐well MASTERBLOCKS (Greiner Bio‐One) with 500 μL of total volume with a range of l‐arabinose concentrations and an equal starting OD600 (0.005, 0.0001 and 0.0000625 for the LeuB‐, KmR‐ or LuxCDABE‐based assays respectively). After growth, 200 μl per culture was transferred to a transparent 96‐well microplate (Greiner Bio‐One, Frickenhausen, Germany) for an OD600 measurement with a Synergy MX microplate reader (BioTek, Winooski, VT, USA). OD600 values were corrected for path length and an average of three blanks. All assays were performed as three independent experiments, being therefore both biological and technical replicates. The data were averaged and the standard deviation was calculated.

For the leucine auxotrophy complementation assays, the two system strains expressing *leuB*, the two positive control strains (non‐auxotrophs with a frameshift in the plasmid encoded *leuB*) and the two negative control strains (auxotrophs with a frameshift in the plasmid encoded *leuB*) were pregrown in minimal M9 medium with 18 μg ml^−1^ of chloramphenicol, 1× minimum essential medium (MEM) vitamins, 20 mg l^−1^ of l‐leucine for complementation and with/without inducer (10 mM of l‐arabinose) for 24 h. In the assays, the leucine concentration was kept below 1 μM to prevent complementation by leucine present in the medium (Sezonov *et al*., 2007). OD600 was measured after 32 and 48 h.

For the kanamycin resistance assays, the two system strains expressing *kan* and the two corresponding negative control strains with a frameshift in *kan* were pregrown in LB medium with 34 μg ml^−1^ of chloramphenicol and with/without inducer (10 mM of l‐arabinose) for 7 h. In the assays, kanamycin concentrations were varied. OD600 was measured after 17 h.

For the bioluminescence assays, the four system strains expressing *lux* and the four corresponding negative control strains with a frameshift in *luxA* were pregrown in LB medium with 34 μg ml^−1^ of chloramphenicol for 17 h. In the assays, OD600 and bioluminescence were measured in the microplate reader after 5.5 h. Bioluminescence was measured in white 96‐well microplates (Thermo Scientific, Nunc; 200 μL per well) under default settings. The temperature of the plate reader was set at 37°C. Bioluminescence values were corrected for the OD600.

### Detection of l‐arabinose isomerase activity

*Geobacillus thermodenitrificans* T12 AraA (GenBank: KX555561) was compared with*. E. coli* MG1655 AraA (GenBank: AAC73173.1) and *G. thermodenitrificans* CBG‐A1 AraA (GenBank: AY302754) by BLASTP 2.3.1+ (Altschul *et al*., 1997, 2005). The plasmids expressing the l‐arabinose isomerases were made in two steps from pWUR873 (GenBank: KX618638), which contained the low copy p15A origin of replication, the ampicillin marker (*bla* encoding β‐lactamase) and the *gpf* gene under control of P_T7_ and a very strong RBS (AAGGAG; −14 to −9 relative to translation start). First, P_T7_ was replaced by the weak to moderate constitutive promoter P_bla_ with KpnI/BcuI, giving pWUR832. The insert was formed by PCR with primers BG4591/BG4304 and pWUR873 as template. Second, the *gfp* CDS was replaced by the *araA* CDS from *E. coli* MG1655 or *G. thermodenitrificans* T12 with NdeI/BcuI, giving pWUR833 and pWUR834 respectively. The inserts were formed by PCR in two steps to remove the NdeI site from the CDS (silent mutation, CAT→CAC). For *araA* of *E. coli*, left and right fragments were created with primers BG6723/BG6726 and BG6725/BG6724 respectively and combined with primers BG6723/BG6724. For *araA* of *G. thermodenitrificans*, left and right fragments were created with primers BG7219/BG7222 and BG7221/BG7220 respectively and combined with primers BG7219/BG7220. A negative control plasmid was formed by making the ends of NdeI/BcuI‐digested pWUR832 blunt with Klenow Fragment and ligating it, giving pWUR917. For verification of the plasmids and the use of enzymes and kits, see section ‘Construction of regulator‐reporter plasmids and control plasmids’. Dephosphorylation was carried out here with fastAP (Thermo Scientific). Strain AR was simultaneously transformed with pWUR768/pWUR833, pWUR768/pWUR834, pWUR768/pWUR917, pWUR780/pWUR833 or pWUR780/pWUR834.

The detection assays were performed as described above for the induction assays, except for a few things. Hundred micrograms per millilitre ampicillin was added to maintain the l‐arabinose isomerase expressing plasmids. Instead of l‐arabinose as inducer of AraC, l‐ribulose was added as substrate for the l‐arabinose isomerase. The l‐ribulose concentration was varied. Cells were not pre‐induced, but in the assay 15 μg ml^−1^ of kanamycin was added after 1 h of growth to allow induction of *kan*. The bioluminescence values were corrected with the values obtained for the negative control with a frameshift in *luxA* (AR with pWUR780/pWUR833 or pWUR780/pWUR834). The kanamycin resistance assay and the bioluminescence assay were performed as two and three independent experiments respectively.

### Enrichment for cells with l‐arabinose isomerase activity

Each of the three strains, AR pWUR768 with pWUR833, pWUR834 or pWUR917, was grown separately in 13 ml LB medium with 4 g l^−1^ of glycerol, 100 μg ml^−1^ of ampicillin and 34 μg ml^−1^ of chloramphenicol. After 24 h, cells were mixed based on the OD600 in a ratio of 1:1:10^8^ for pWUR833:pWUR834:pWUR917 and grown in 25 ml of the same medium with the addition of 15 μg ml^−1^ of kanamycin as selective pressure and 5 mM of l‐ribulose as substrate for the l‐arabinose isomerase. The controls were 1 ml of culture with l‐ribulose and with/without kanamycin inoculated with each of the strains separately. After 6 h, dilution series were streaked on three types of LB agar plates with 100 μg ml^−1^ of ampicillin and 34 μg ml^−1^ of chloramphenicol, namely (i) without either l‐ribulose or kanamycin, (ii) with 15 μg ml^−1^ of kanamycin and (iii) with both 15 μg ml^−1^ of kanamycin and 5 mM of l‐ribulose. Colonies were counted and 68 individual colonies, originating from the plates with kanamycin and l‐ribulose that were inoculated with the mixed culture, were picked for the subsequent bioluminescence‐based screen. White 96‐well microplates (Thermo Scientific) with 200 μl LB medium per well with 15 g l^−1^ of agar, 100 μg ml^−1^ of ampicillin, 34 μg ml^−1^ of chloramphenicol and 0 or 0.5 mM of l‐ribulose, were inoculated with one colony per well. After 17 h growth, bioluminescence was detected with the lumiglo function of the G:BOX Chemi XT4 (Syngene, Cambridge, United Kingdom). Biomass from these plates was used as template in several PCRs to show the presence or absence of l‐arabinose isomerase genes (primers BG3799/BG6225), identity of l‐arabinose isomerase genes (primers BG7642/7643/7644) or occurrence of *araC*‐*kan* CDS exchange (primers BG7009/4588/3652). PCRs were performed with OneTaq (NEB) and primers are presented in Table S4. The *araC*‐*kan* CDS exchange was analysed by sequencing at GATC Biotech.

## Conflicts of interest

None declared.

## Supporting information


**Fig. S1.** Overview cloning steps.
**Fig. S2.** Effect of the addition of inducer to the preculture on the leucine auxotrophy complementation assay.
**Fig. S3.** Selection based on leucine auxotrophy complementation (48 h).
**Fig. S4.** Effect of the addition of inducer to the preculture on the kanamycin resistance assay.
**Fig. S5.** Kanamycin death curve.
**Fig. S6.** Growth of cells with or without l‐arabinose isomerase (*araA*).
**Fig. S7.** l‐arabinose isomerase (AraA) expression analysis.
**Fig. S8.** Bioluminescence‐based screening after enrichment of l‐arabinose isomerase (*araA)* containing cells.
**Table S1.** Frameshifts in control plasmids.
**Table S2.** Relative plasmid copy number of the reporter systems.
**Table S3.** Sequences.
**Table S4.** Primers used in this study.
