# Supplementary material for: A growth‐ and bioluminescence‐based bioreporter for the in vivo detection of novel biocatalysts
Source: Microb Biotechnol. 2017 Apr 10;10(3):625–41. doi: 10.1111/1751-7915.12612 (PMC5404197; doi:10.1111/1751-7915.12612)
Supplement: Supplementary file 1 — Fig. S1. Overview cloning steps. Fig. S2. Effect of the addition of inducer to the preculture on the leucine auxotrophy complementation assay. Fig. S3. Selection based on leucine auxotrophy complementation (48 h). Fig. S4. Effect of the addition of inducer to the preculture on the kanamycin resistance assay. Fig. S5. Kanamycin death curve. Fig. S6. Growth of cells with or without l‐arabinose isomerase (araA). Fig. S7. l‐arabinose isomerase (AraA) expression analysis. Fig. S8. Bioluminescence‐based screening after enrichment of l‐arabinose isomerase (araA) containing cells. Table S1. Frameshifts in control plasmids. Table S2. Relative plasmid copy number of the reporter systems. Table S3. Sequences. Table S4. Primers used in this study. [file MBT2-10-625-s001.docx]

**Supporting Information**

A Growth- and Bioluminescence-Based Bioreporter for the In Vivo Detection of Novel Biocatalysts

Teunke van Rossum^1*^, Aleksandra Muras^1^, Marco J.J. Baur^1^, Sjoerd C.A. Creutzburg^1^, John van der Oost^1^, Servé W.M. Kengen^1**^

^1^ Laboratory of Microbiology, Wageningen University and Research, Stippeneng 4, 6708 WE Wageningen, The Netherlands

For correspondence

*Email: teunke.vanrossum@wur.nl, Phone: +31 317 481066

**Email: serve.kengen@wur.nl, Phone: +31 317 483737

**Table S1. Frameshifts in control plasmids*^a^***

| **Construct** | **Parent*^b^*** | **Expected*^c^*** | **Obtained*^d^*** | **Comments** |
| --- | --- | --- | --- | --- |
| pWUR778/80/86/88 | CCG**CG**G | CCGG | CCGG | as expected*^e^* |
| (*luxA)* | G**GC**GCC | GGCC | GGCC | residues 1-160 ok, A161G, A162V, Y163Stop (parent: 361 codons)*^f^* |
| pWUR774 (*leuB*) | GACAT**TC**TGTC | GACATCTGTC | GACATTGTC | 2 bp instead of 1 bp deletion (stop 5 codons earlier, also 252 codons ok, C253V, L254Stop)*^e^* |
|  | CTGT**AA**GACAG | CTGTAGACAG | CTGTAACAG | residues 1-252 ok, L253V, S254Stop (parent: 364 codons)*^f^* |
| pWUR782 (*leuB*) | GACAT**TC**TGTC | GACATCTGTC | GACACTGTC | 2 bp instead of 1 bp deletion (stop 5 codons earlier, 251 instead of 252 codons ok, I252T, |
|  | CTGT**AA**GACAG | CTGTAGACAG | CTGTGACAG | C253V, L254Stop)*^e^* |
|  |  |  |  | residues 1-251 ok, I252T, L253V, S254Stop (parent: 364 codons)*^f^* |
| pWUR776 (*kan*) | CCTG**AT**TCAGG | CCTGATTTCAGG | CCTGATCAGG | 1 bp deletion instead of 1 bp insertion (stop 38 codons later, also 115 codons ok)*^e^* |
|  | GGACT**AA**GTCC | GGACTAAAGTCC | GGACTAGTCC | residues 1-115 ok, S116Q, G117V, E118K, ………., R155E, M156Stop (parent: 272 codons)*^f^* |
| pWUR784 (*kan*) | CCTG**AT**TCAGG | CCTGATTTCAGG | CCTGATTTCAGG | as expected*^e^* |
|  | GGACT**AA**GTCC | GGACTAAAGTCC | GGACTAAAGTCC | residues 1-115 ok, S116F, G117R, E118Stop (parent: 272 codons)*^f^* |

*^a^*All upper strands are from 5’ to 3’ in the direction of the gene indicated in brackets. *^b^*The restriction site in the parent plasmid (Cfr42I, Eam1105I and XagI for *luxA*, *leuB* and *kan*, respectively). The cut site is between the two bold nucleotides. *^c^*Sequence expected after Klenow treatment and ligation. *^d^*Sequence obtained after Klenow treatment and ligation. *^e^*Obtained versus expected. For the *luxA* gene, the obtained sequence of the frameshift site conformed to the expected fill in and removal of 5’ and 3’overhangs, respectively. Surprisingly, the sequence in the *kan* and *leuB* genes differed from this expectation, but this difference can be explained by the GC-content of the termini after digestion. Both *kan* and *leuB* genes had AT-rich termini, whereas *luxA* had GC-rich termini. AT-rich termini make partitioning of the strands easier than GC-rich termini, increasing the preference of the DNA to be bound in the exonuclease domain of Klenow ([Carver *et al.*, 1994](#_ENREF_3)). Increased 3’ to 5’exonuclease activity compared to 5’ to 3’ polymerase activity explained not only the deviation in the sequence, but it also explained the variation between different constructs made with the same restriction enzyme. The overhangs probably mismatched, but were ligated anyway and later repaired by *E. coli* using either one or the other strand as template, giving rise to two different sequences. Despite these differences between controls made with the same restriction enzyme, altered truncated products did not change the signal (growth or bioluminescence) of these negative controls (see characterization section in main text). *^f^*Obtained versus parent.

**Table S2. Relative plasmid copy number of the reporter systems**

| **Strain*^a^*** | **10^9^ plasmids mL^-1^*^b^*** | | **Ratio medium/low^c^** |
| --- | --- | --- | --- |
|  | low copy | medium copy |  |
| ALR + reg.-rep. plasmid (*araC*, *leuB*) | 5.7 ± 0.6 | 24.7 ± 3.7 | 4.4 ± 0.8 |
| AR + reg.-rep. plasmid (*araC*, *kan*) | 5.5 ± 2.0 | 28.2 ± 1.6 | 5.1 ± 1.8 |
| ALR + control plasmid (*araC*, *leuB*−) | - | 26.0 ± 1.2 | - |
| AR + control plasmid (*araC*, *kan*−) | - | 26.4 ± 2.3 | - |
| AR + control plasmid (*araC*, *kan, luxA*−) | - | 24.2 ± 2.1 | - |
| DH10B + reg.-rep. plasmid (*araC*, *kan*) | 11.9 ± 4.6 | 55.8 ± 21.4 | 4.7 ± 2.6 |
| DH10B + pACYC184 | 20.8 ± 5.0 | - | - |

*^a^*The systems vary in the selection reporter (LeuB or KmR) and the copy number of the regulator-reporter plasmid (ColE1 or p15A origins of replications for medium or low copy number, respectively). The control plasmids have a frameshift in one of the reporter genes, indicated with a minus. The controls for plasmid isolation are another plasmid with the p15A origin of replication (pACYC184 ([Chang and Cohen, 1978](#_ENREF_4))) and a control strain adapted for cloning (*E. coli* DH10B T1^R^). reg.-rep. plasmid, regulator-reporter plasmid. ^b^The number of plasmid molecules per milliliter culture at an OD600 of 1 was determined by plasmid isolation. All strains were grown overnight in 10 mL LB medium with the appropriate antibiotics. After OD600 measurement, plasmids were isolated with the Plasmid Miniprep kit of Thermo Scientific (#K0503). Plasmid concentration was measured with a ND-1000-Spectrophotometer (NanoDrop Technologies, Inc.). The total amount of isolated plasmid in micrograms was corrected for the amount of supernatant loaded on the column and divided by the OD600 and the culture volume. The negative control value (no plasmid) was subtracted. The number of plasmids per mL culture with OD600 = 1 was obtained by converting this total plasmid weight to the number of plasmid molecules (650 g mol^-1^ bp^-1^). The data are an average of three independent experiments (standard deviation indicated). The ratio between frameshift control and parent plasmid was 1.0, confirming the expected similarity between the controls and their parent plasmids. pACYC184 is 2.5 times smaller than the regulator-reporter plasmids, but it also has the low copy origin of replication (p15A). pACYC184 had a ~2 higher copy number than the regulator-reporter plasmid with p15A. This inverse relation between plasmid size and copy number has also been described by others ([Bron and Luxen, 1985](#_ENREF_1)). All tested plasmids had a two times higher copy number in the cloning strain *E. coli* DH10B T1^R^ than in the *E. coli* BW25113 knockout strains. This twofold difference is probably due to the adaptation of *E. coli* DH10B T1^R^ for cloning and thus for elevated plasmid yields.

**Table S3. Sequences.**

1. P_BAD-adapt_ in front of *leuB*/*kan*. Underlined and italic nucleotides indicate restriction sites and operator (half) sites (ecogene.org, Invitrogen pBAD ([Dunn and Schleif, 1984](#_ENREF_5), [Carra and Schleif, 1993](#_ENREF_2))) respectively. The sequence contains from 5’ to 3’ an XmaJI site, an O2 operator half site (AraC), an O1 operator half site (AraC), an O1 operator half site (AraC), a randomized CRP binding site including an NheI site, an I1 operator half site (AraC), an I2 operator half site (AraC), the promoter -35 site, the promoter -10 site, and an MreI site.

CCTAGGGCCATTCAGAGAA*GAAACCAATTGTCCATA*TTGCATCAGACATTGCCGTCACTGCGTCTTTTACTGGCTCTTCTCGCTAACCAAACCGGTAACCCCGCTTATTAAAAGCATTCTGTAACAAAGCGGGACCAAAGCCATGACAAAAACGCG*TAACAAAAGTGTCTATA*ATCA*CGGCAGAAAAGTCCACA*TTGATTA*ACCTAAGTCGAGATGGAAGCTAGCTCG*CA*TAGCATTTTTATCCATA*AGAT*TAGCGGATCCTACCTGA*CGCTTTTTATCGCAACTCTCTACTGTTTCTCCATACCCGTTTTTTTGGGCGCCGGCG

1. P_BAD-adapt_ in front of *luxCDABE*. Underlined and italic nucleotides indicate restriction sites and operator (half) sites (ecogene.org, Invitrogen pBAD ([Dunn and Schleif, 1984](#_ENREF_5), [Carra and Schleif, 1993](#_ENREF_2))) respectively. The sequence contains from 5’ to 3’ an Acc65I site, an O2 operator half site (AraC), an O1 operator half site (AraC), an O1 operator half site (AraC), a randomized CRP binding site including a PstI site, an I1 operator half site (AraC), an I2 operator half site (AraC), the promoter -35 site, the promoter -10 site, and a SalI site.

GGTACCGCCATTCAGAGAA*GAAACCAATTGTCCATA*TTGCATCAGACATTGCCGTCACTGCGTCTTTTACTGGCTCTTCTCGCTAACCAAACCGGTAACCCCGCTTATTAAAAGCATTCTGTAACAAAGCGGGACCAAAGCCATGACAAAAACGCG*TAACAAAAGTGTCTATA*ATCA*CGGCAGAAAAGTCCACA*TTGATTA*ACCTAAGTCGAGATGGAACTGCAGTCG*CA*TAGCATTTTTATCCATA*AGAT*TAGCGGATCCTACCTGA*CGCTTTTTATCGCAACTCTCTACTGTTTCTCCATACCCGTTTTTTTGGGGTCGAC

1. Disruption cassette. Underlined and italic nucleotides indicate restriction sites and *lox* sites, respectively. The cassette contains from 5’to 3’ a SfiI site (used for cloning by GeneArt), a HindIII site, a site for primer annealing, a PstI site, *lox*71, a BglII site, *kan*, a SalI site, *lox*66, a NotI site, a site for primer annealing, an EcoRI site, and a SfiI site (used for cloning by GeneArt).

GGCCGTCAAGGCCGCATAAGCTTGGTGTCTTTTTTACCTGTTTGACCCTGCAG*TACCGTTCGTATAATGTATGCTATACGAAGTTAT*AGATCTCTATTTGTTTATTTTTCTAAATACATTCAAATATGTATCCGCTCATGAGACAATAACCCTGATAAATGCTTCAATAATATTGAAAAAGGAAGAGTATGAGCCATATTCAACGGGAAACGTCTTGCTCTAGGCCGCGATTAAATTCCAACATGGATGCTGATTTATATGGGTATAAATGGGCTCGCGATAATGTCGGGCAATCAGGTGCGACAATCTATCGATTGTATGGGAAGCCCGATGCGCCAGAGTTGTTTCTGAAACATGGCAAAGGTAGCGTTGCCAATGATGTTACAGATGAGATGGTCAGACTAAACTGGCTGACGGAATTTATGCCTCTTCCGACCATCAAGCATTTTATCCGTACTCCTGATGACGCATGGTTACTCACCACTGCGATCCCCGGGAAAACAGCATTCCAGGTATTAGAAGAATATCCTGATTCAGGTGAAAATATTGTTGATGCGCTGGCAGTGTTCCTGCGCCGGTTGCATTCGATTCCTGTTTGTAATTGTCCTTTTAACAGCGACCGCGTATTTCGTCTCGCTCAGGCGCAATCACGAATGAATAACGGTTTGGTTGATGCGAGTGATTTTGATGACGAGCGTAATGGCTGGCCTGTTGAACAAGTCTGGAAAGAAATGCACAAACTTTTGCCATTCTCACCGGATTCAGTCGTCACTCATGGTGATTTCTCACTTGATAACCTTATTTTTGACGAGGGGAAATTAATAGGTTGTATTGATGTTGGACGAGTCGGAATCGCAGACCGATACCAGGATCTTGCCATCCTATGGAACTGCCTCGGTGAGTTTTCTCCTTCATTACAGAAACGGCTTTTTCAAAAATATGGTATTGATAATCCTGATATGAATAAATTGCAGTTTCATTTGATGCTCGATGAGTTTTTCTAAGTCGAC*ATAACTTCGTATAATGTATGCTATACGAACGGTA*GCGGCCGCCAACTCCTTCACCAGAGGTAGGAATTCCTGGGCCTCATGGGCC

**Table S4. Primers used in this study**

| **Primer** | **Annealing location** | **Sequence (5'-->3')^a^** | **Features** |
| --- | --- | --- | --- |
| ***Construction of plasmids*** | | | |
| BG3691 | start *kan* | GCGCGGGATCCCGCCGGCG*AGGAGG*ATACGTATGAGCCATATTCAACGGGAAAC | BamHI, MreI, RBS |
| BG3692 | end *kan* | CGCGCCTCGAGCGATCGTTAGAAAAACTCATCGAGCATCAAATG | XhoI, PvuI |
| BG3693 | start *leuB* | GCGCGGGATCCCGCCGGCG*AGGAGG*ATACGTATGTCGAAGAATTACCATATTGCCG | BamHI, MreI, RBS |
| BG3694 | end *leuB* | CGCGCCTCGAGCGATCGTTACACCCCTTCTGCTACATAGC | XhoI, PvuI |
| BG3695 | inside *leuB* | GAATCTGCTCGCAAGCGTCGCCACAAAGTGACG*AGT*ATCGATAAAGCCAAC | altered serine codon: TCG-->AGT |
| BG3696 | inside *leuB* | GAGGATTGCAGCACGTTGGCTTTATCGAT*ACT*CGTCACTTTGTGGCGACG | altered serine codon: TCG-->AGT |
| BG3746 | start P_lacI_^Q^ | GCGCGCTCGAGGTTGACACCATCGAATGGTGCAAAACC | XhoI |
| BG3747 | end P_lacI_^Q^ | GCCGCCGATCGCGGWCCGATTCACCACCCTGAATTGACTCTCTTCC | PvuI, CpoI |
| BG3940 | start *araC* | GCGCGCGGWCCG*AGGAGG*ATACGTATGGCTGAAGCGCAAAATGATC | CpoI, RBS |
| BG3941 | end *araC* | CGCGCCGATCGTTATGACAACTTGACGGCTACATC | PvuI |
| BG3979 | start P_BAD_ | GCCGGGGTACCGCCATTCAGAGAAGAAACC | KpnI |
| BG3980 | end P_BAD_ | CCGGCGTCGACCCCAAAAAAACGGGTATGG | SalI |
| BG3981 | end P_BAD_ | CTCTTCGCCGGCGCCCAAAAAAACGGGTATGG | MreI |
| BG3982 | inside P_BAD_ | *CCTAAGTCGAGATGGAACTGCAGTCG*CATAGCATTTTTATCCATAAGATTAGC | altered CRP binding site, PstI |
| BG3983 | inside P_BAD_ | CGA*CTGCAGTTCCATCTCGACTTAGGT*TAATCAATGTGGACTTTTCTGC | altered CRP binding site, PstI |
| BG4229 | inside P_BAD_adapt_ | G*CGAGCTAGCTTCCATCTCGACTTAGGT*TAATC | NheI, altered CRP binding site |
| BG4230 | inside P_BAD_adapt_ | *GGAAGCTAGCTCG*CATAGCATTTTTATCCATAAG | part altered CRP binding site, NheI |
| BG4231 | start ColE1 | TACTGGTACCCATGACCAAAATCCCTTAACGTG | Acc65I |
| BG4232 | end ColE1 | TACTCGTACGCCTAGGCGTTCGGCTGC | Pfl23II |
| BG4304 | end *gfp* | TACTACTAGTTTATTTGTAGAGCTCATCCATGCCATGTG | BcuI |
| BG4368 | start P_BAD_ | CTACTCCTAGGGCCATTCAGAGAAGAAACC | XmaJI |
| BG4591 | start *gfp* | TACTGGTACCCCGCTTCGGCGGGGTTTTTTCAAGTTCAAATATGTATCCGCTCATGAGACAATGTGTGGG  GAGACCACAACGGTTTCC | KpnI |
| BG4666 | start p15A | TATGTGGTACCTAGCGGAGTGTATACTGGCTTAC | Acc65I |
| BG4667 | end p15A | TACAACCTAGGACAACTTATATCGTATGGGGCTG | XmaJI |
| BG6723 | start *araA*^b^ | CGCGCCATATGACGATTTTTGATAATTATGAAGTGTGG | NdeI |
| BG6724 | end *araA*^b^ | GCGCGACTAGTTTAGCGACGAAACCCG | BcuI |
| BG6725 | inside *araA*^b^ | GGTGCTCGGCTCC*CAC*ATGCTGGAAGTCTGC | altered histidine codon: CAT-->CAC |
| BG6726 | inside *araA*^b^ | GCAGACTTCCAGCAT*GTG*GGAGCCGAGCACC | altered histidine codon: CAT-->CAC |
| BG7219 | start *araA*^c^ | CGCGCCATATGTTATCATTACGTCCTTATGAATTTTGG | NdeI |
| BG7220 | end *araA*^c^ | GCGCGACTAGTTTACCTCCCTCGCCAAAATAC | BcuI |
| BG7221 | inside *araA*^c^ | GATTCTTGGCGCT*CAC*ATGCTCGAAGTATGC | altered histidine codon: CAT-->CAC |
| BG7222 | inside *araA*^c^ | GCATACTTCGAGCAT*GTG*AGCGCCAAGAATC | altered histidine codon: CAT-->CAC |

**Table S4 continued**

| **Primer** | **Annealing location** | **Sequence (5'-->3')** | **Features** |
| --- | --- | --- | --- |
| ***Verification of plasmids*** | | | |
| BG3336 | inside ColE1 | TTCGCCACCTCTGACTTG |  |
| BG3652 | inside *kan* | AGTAACCATGCGTCATCAGG |  |
| BG3653 | inside *kan* | GCCTGTTGAACAAGTCTGGA |  |
| BG3799 | inside p15A | CAGAGCAAGAGATTACGCGCAGACC |  |
| BG3857 | inside *luxE* | GAAGCGTTTGATAGTTGAGCGG |  |
| BG3858 | inside *cat* | CAGGTTCATCATGCCGTCTG |  |
| BG3942 | upstream *cat* | CAACGTCTCATTTTCGCCAG |  |
| BG3943 | inside *luxC* | CACGAATGTATGTCCTGCG |  |
| BG3977 | inside *leuB* | GCACAAATCCTTTCGCTGG |  |
| BG3978 | inside *leuB* | GTAATGGCTGGTGGTGATG |  |
| BG4231 | start ColE1 | TACTGGTACCCATGACCAAAATCCCTTAACGTG | Acc65I |
| BG4232 | end ColE1 | TACTCGTACGCCTAGGCGTTCGGCTGC | Pfl23II |
| BG4627 | inside *leuB* | GTATTCCGTGGCGATCTC |  |
| BG4628 | inside *leuB* | CGGCATCTATTTCGGTCAG |  |
| BG4629 | inside *kan* | CCAGACTTGTTCAACAGGC |  |
| BG4630 | inside *kan* | CTCCTGATGACGCATGG |  |
| BG4631 | inside *luxA* | GAATGGCATGACAGAGGG |  |
| BG4632 | inside *luxA* | GTGCCCATATTCTTGAGCC |  |
| BG6225 | inside T7 terminator | CCTCAAGACCCGTTTAGAGG |  |
| ***Construction of knockouts*** | | | |
| BG3649 | start cassette^d^ | *GCTCAACACAACGAAAACAACAAGGAAACCGTGTGA*GGTGTCTTTTTTACCTGTTTGACC | homologous to genome (upstream/start *leuB)* |
| BG3650 | end cassette^d^ | *ACGTCTTAGCCATGATTACACCCCTTCTGCTACATA*CTACCTCTGGTGAAGGAGTTG | homologous to genome (end/downstream *leuB*) |
| BG4490 | start cassette^d^ | *CAGAACATATTGACTATCCGGTATTACCCGGCATGACAGGAGTAAAAATG*GGTGTCTTTTTTACCTGTTTGACC | homologous to genome (upstream/start *recA*) |
| BG4491 | end cassette^d^ | *ATGCGACCCTTGTGTATCAAACAAGACGATTAAAAATCTTCGTTAGTTTC*CTACCTCTGGTGAAGGAGTTG | homologous to genome (end/downstream *recA*) |
| ***Verification of knockouts*** | | | |
| BG3651 | upstream *leuB* | CAGGTGGATATCGTCGCTAA |  |
| BG3652 | inside *kan* | AGTAACCATGCGTCATCAGG |  |
| BG3653 | inside *kan* | GCCTGTTGAACAAGTCTGGA |  |
| BG3654 | downstream *leuB* | AACAGTGGGGTTTCGTTTTC |  |
| BG3655 | upstream *araC* | GGTTGGGTTAGCGAGAAGAG |  |
| BG3656 | downstream *araC* | GGGTAGAATCAAACCGACCA |  |
| BG4190 | upstream *recA* | CGTCAGGCTACTGCGTATG |  |
| BG4191 | downstream *recA* | GAATACGCGCAGGTCCATAAC |  |

**Table S4 continued**

| **Primer** | **Annealing location** | **Sequence (5'-->3')** | **Features** |
| --- | --- | --- | --- |
| ***Analysis of enriched colonies*** | |  |  |
| BG3652 | inside *kan* | AGTAACCATGCGTCATCAGG |  |
| BG3799 | inside p15A | CAGAGCAAGAGATTACGCGCAGACC |  |
| BG4588 | inside *araC* | TACTGACAAGCCTCGCGTACCC |  |
| BG6225 | inside T7 terminator | CCTCAAGACCCGTTTAGAGG |  |
| BG7009 | inside CmR | GGTTATAGGTACATTGAGCAACTG |  |
| BG7642 | inside *araA*^b,c^ | CCGTGGGACAGCATCGATATGG |  |
| BG7643 | inside *araA*^b^ | GCCCTGCAGACCGGTTG |  |
| BG7644 | inside *araA*^c^ | GCTTTGTTCAGAAACATCGCGAATAG |  |

^a^Underlined nucleotides indicate restriction sites, italic nucleotides indicate other features (for both see last column). ^b^*araA* of *E. coli*. ^c^*araA* of *G. thermodenitrificans*. ^d^Recombination/disruption cassette.

**Fig. S1. Overview cloning steps.** The four regulator-reporter plasmids (depicted in bold with a grey background) were constructed from pFU98 ([Uliczka *et al.*, 2011](#_ENREF_6)) in eight subsequent cloning steps. From each of the four plasmids two control plasmids were made (depicted with a grey background) by making a frameshift either in the selection reporter gene (*leuB*/*kan*) or in one gene in the screening reporter operon (*luxA*). For each cloning step a description and the restriction enzymes are included. Rep., replacement; Ins., insertion; Trans., translocation; Fram., introduction frameshift. The origin and formation of the inserts are described here. BG numbers refer to primers (Table S4). pWUR749: digest from pFU168 ([Uliczka *et al.*, 2011](#_ENREF_6)). pWUR750: two step PCR from BW25113 genome to remove AatII and PvuI sites in CDS (first left BG3693/BG3696, first right BG3695/BG3694, second BG3693/BG3694). pWUR751: PCR from recombination cassette ([Westra *et al.*, 2010](#_ENREF_7)) (BG3691/BG3692). pWUR752/3: PCR from pET24d (Novagen, BG3746/BG3747). pWUR754/6: PCR from pBAD-TOPO (Invitrogen, BG3940/BG3941). pWUR758/60: two step PCR from pBAD-TOPO with adaptations (first left BG3979/BG3983, first right BG3982/BG3980, second BG3979/BG3980). pWUR762/4: PCR from pWUR758 (BG4232/BG4231). pWUR766/8: two PCRs from pWUR758 with adaptations (BG4368/BG4229, BG4230/BG3981). pWUR770/2: PCR from pACYC184([Chang and Cohen, 1978](#_ENREF_4)) (BG4666/BG4667). pWUR786/8: digest from pWUR778.

**Fig. S2. Effect of the addition of inducer to the preculture on the leucine auxotrophy complementation assay.** The plasmid-encoded reporter gene *leuB* was induced or non-induced in the low and medium copy systems with 10 mM (no full induction) of the inducer L-arabinose. The cultures were inoculated from induced or non-induced precultures to see the effect of this treatment. “−/−” non-induced in both precultures and assay cultures, “−/+” induced only in assay cultures, “+/+” induced in both precultures and assay cultures. Bacteria were grown in M9 medium for 32 h. The data are an average of three independent experiments (standard deviation indicated). System: auxotroph *E. coli* BW25113 Δ*araC* Δ*leuB* Δ*recA* (ALR) with the regulator-reporter plasmid. Neg. ctrl.: auxotroph ALR with the regulator-reporter plasmid with a frameshift in *leuB*. Pos. ctrl.: non-auxotroph *E. coli* BW25113 Δ*araC* Δ*recA* (AR) with the regulator-reporter plasmid with a frameshift in *leuB*.

**Fig. S3. Selection based on leucine auxotrophy complementation (48 h).** The plasmid-encoded reporter gene *leuB* was induced in the low and medium copy systems with the inducer L-arabinose. Bacteria were grown in M9 medium for 48 h. The data are an average of three independent experiments (standard deviation indicated). System: auxotroph *E. coli* BW25113 Δ*araC* Δ*leuB* Δ*recA* (ALR) with the regulator-reporter plasmid. Neg. ctrl.: auxotroph ALR with the regulator-reporter plasmid with a frameshift in *leuB*. Pos. ctrl.: non-auxotroph *E. coli* BW25113 Δ*araC* Δ*recA* (AR) with the regulator-reporter plasmid with a frameshift in *leuB*.

**Fig. S4. Effect of the addition of inducer to the preculture on the kanamycin resistance assay.** The plasmid-encoded reporter gene *kan* was induced or non-induced in the low and medium copy systems with 10 mM (no full induction) of the inducer L-arabinose. The cultures were inoculated from induced or non-induced precultures to see the effect of this treatment. “−/−” non-induced in both precultures and assay cultures, “−/+” induced only in assay cultures, “+/+” induced in both precultures and assay cultures. Bacteria were grown in LB medium for 17 h in presence of 0, 10 or 20 μg mL^-1^ kanamycin. The data are an average of three independent experiments (standard deviation indicated). System: *E. coli* BW25113 Δ*araC* Δ*recA* (AR) with the regulator-reporter plasmid. Neg. ctrl.: AR with the regulator-reporter plasmid with a frameshift in *kan*.

**Fig. S5. Kanamycin death curve.** The plasmid-encoded reporter gene *kan* was induced “+” or non-induced “−” in the low “L” and medium “M” copy systems with 10 mM (no full induction) of the inducer L-arabinose. Bacteria were grown in LB medium for 17 h in presence of different kanamycin concentrations. The data are an average of three independent experiments (standard deviation indicated). System: *E. coli* BW25113 Δ*araC* Δ*recA* (AR) with the regulator-reporter plasmid. Neg. ctrl.: AR with the regulator-reporter plasmid with a frameshift in *kan*.

**Fig. S6. Growth of cells with or without L-arabinose isomerase (*araA*).** Bacteria were grown in LB medium for 5.5 h in presence of a range of L-ribulose (substrate) concentrations. *araA E. coli*: *E. coli* BW25113 Δ*araC* Δ*recA* (AR) with the regulator-reporter plasmid and the plasmid expressing *araA* of *E. coli*. *araA G. therm*: AR with the regulator-reporter plasmid and the plasmid expressing *araA* of *G. thermodenitrificans*. Empty plasmid: AR with the regulator-reporter plasmid and the empty plasmid.

**
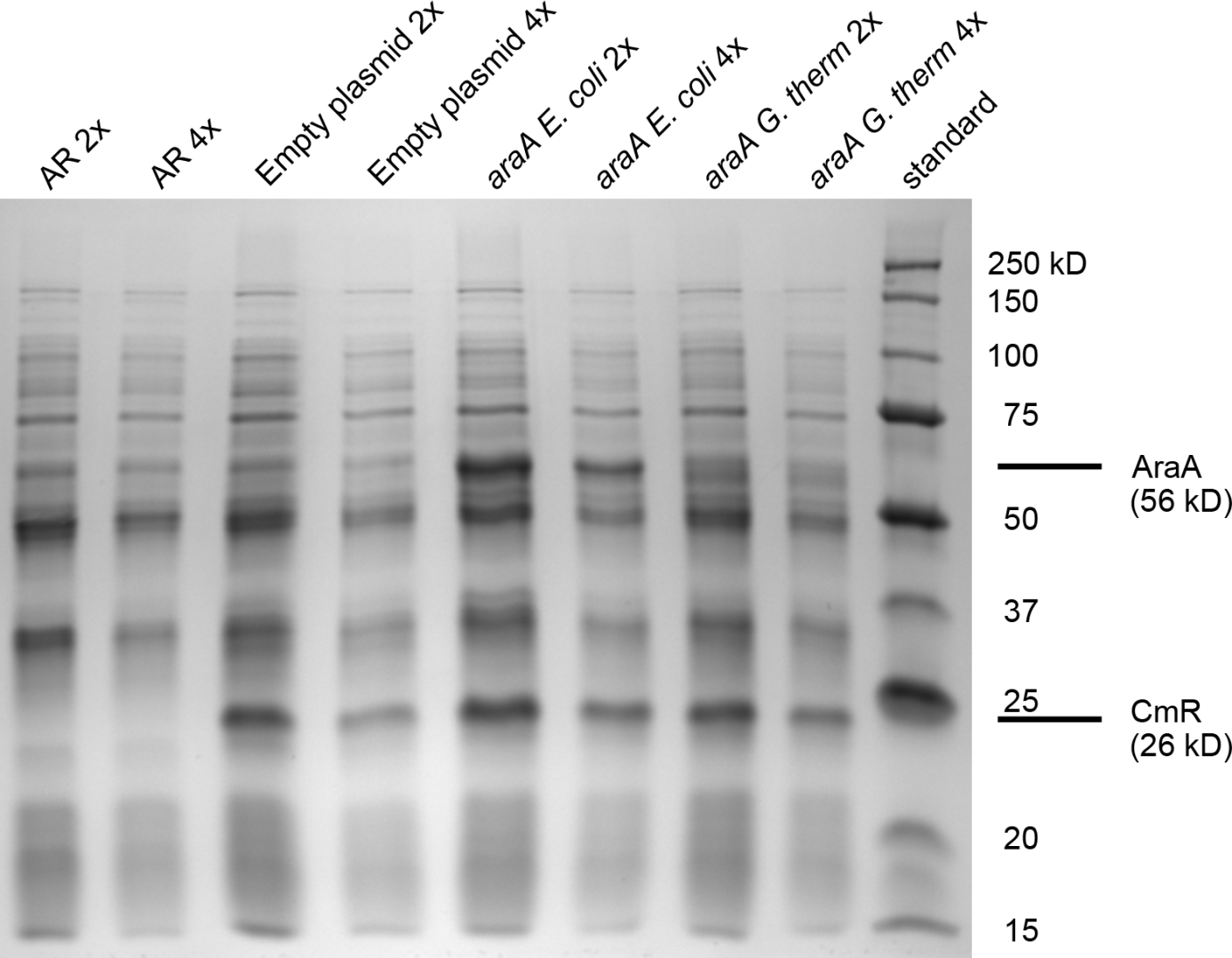
**

**Fig. S7. L-arabinose isomerase (AraA) expression analysis.** Three mL LB medium with 100 µg mL^-1^ ampicillin and 34 µg mL^-1^ chloramphenicol were inoculated with one of the four strains (strain without plasmids was grown without antibiotics). After 19 h growth, an equivalent of 1 mL of cells of OD600 = 0.8 was centrifuged. The pellet was resuspended in 1x Laemmli Sample Buffer (Biorad) including beta-mercaptoethanol. Two and four times diluted samples were boiled for 15 min at 98°C and, after centrifugation, 10 µl per sample and 10 µl of Precision Plus Protein Unstained Standard (Biorad) were loaded on a 10% SDS-PAGE Mini-PROTEAN TGX Precast Protein Gel (Biorad). The gel was run at 20 mA in 25 mM Tris, 192 mM glycine and 0.1% SDS. Proteins were fixed with 25% isopropanol and 10% acetic acid for 15 minutes and stained with QC Colloidal Coommassie Stain (Biorad) overnight. The gel was destained with milliQ for 3 h. *araA E. coli*: *E. coli* BW25113 Δ*araC* Δ*recA* (AR) with the regulator-reporter plasmid (pWUR768) and the plasmid expressing *araA* of *E. coli* (pWUR833). *araA G. therm*: AR with the regulator-reporter plasmid (pWUR768) and the plasmid expressing *araA* of *G. thermodenitrificans* (pWUR834). Empty plasmid: AR with the regulator-reporter plasmid (pWUR768) and the empty plasmid (pWUR917). AR: *E. coli* BW25113 Δ*araC* Δ*recA* without plasmids. 2x and 4x indicate the dilution and CmR indicates the chloramphenicol resistance marker on the regulator-reporter plasmid.

**
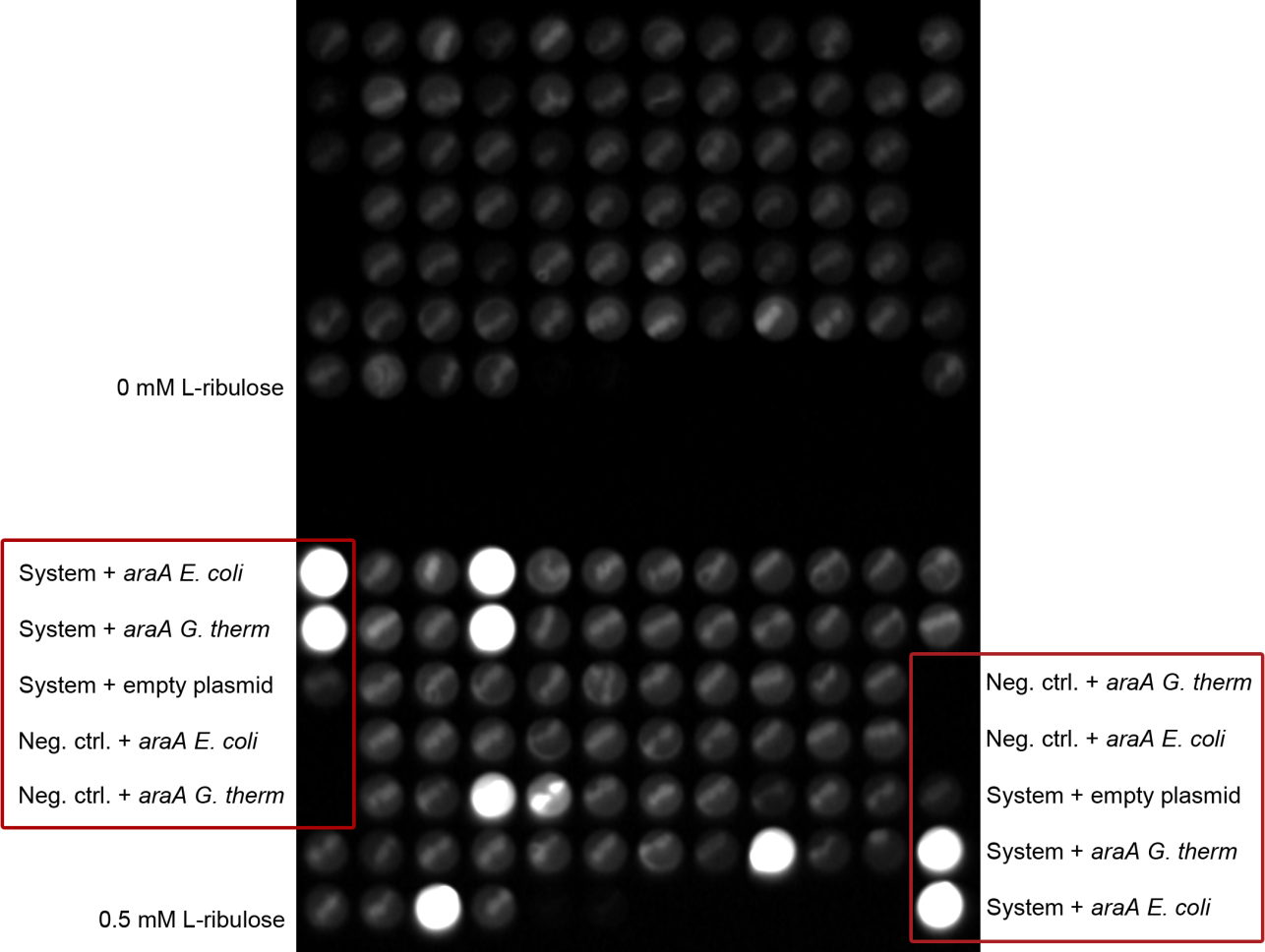
**

**Fig. S8 Bioluminescence-based screening after enrichment of L-arabinose isomerase (*araA*) containing cells.** Sixty eight colonies of the enrichment were tested for L-ribulose dependent bioluminescence to confirm presence of *araA*. Conversion of L-ribulose to L-arabinose by the L-arabinose isomerase of *E. coli* or *G. thermodenitrificans* induced the system. Bacteria were grown in a 96-well plate on LB medium with agar with (bottom) or without (top) 0.5 mM L-ribulose for 17 h. The five controls were included twice. System + *araA E. coli*: *E. coli* BW25113 Δ*araC* Δ*recA* (AR) with the regulator-reporter plasmid and the plasmid expressing *araA* of *E. coli*. System + *araA G. therm*: AR with the regulator-reporter plasmid and the plasmid expressing *araA* of *G. thermodenitrificans*. System + empty plasmid: AR with the regulator-reporter plasmid and the empty plasmid. Neg. ctrl. + *araA E. coli*: AR with the regulator-reporter plasmid with a frameshift in *luxA* and the plasmid expressing *araA* of *E. coli*. Neg. ctrl. + *araA G. therm*: AR with the regulator-reporter plasmid with a frameshift in *luxA* and the plasmid expressing *araA* of *G. thermodenitrificans*.

**References**

Bron, S., and Luxen, E. (1985) Segregational instability of pUB110-derived recombinant plasmids in *Bacillus subtilis*. *Plasmid* **14**: 235-244.

Carra, J.H., and Schleif, R.F. (1993) Variation of half-site organization and DNA looping by AraC protein. *EMBO J.* **12**: 35-44.

Carver, T.E., Hochstrasser, R.A., and Millar, D.P. (1994) Proofreading DNA: recognition of aberrant DNA termini by the Klenow fragment of DNA polymerase I. *Proc. Natl. Acad. Sci. U. S. A.* **91**: 10670-10674.

Chang, A.C., and Cohen, S.N. (1978) Construction and characterization of amplifiable multicopy DNA cloning vehicles derived from the P15A cryptic miniplasmid. *J. Bacteriol.* **134**: 1141-1156.

Dunn, T.M., and Schleif, R. (1984) Deletion analysis of the *Escherichia coli* *ara* P_C_ and P_BAD_ promoters. *J. Mol. Biol.* **180**: 201-204.

Uliczka, F., Pisano, F., Kochut, A., Opitz, W., Herbst, K., Stolz, T., and Dersch, P. (2011) Monitoring of gene expression in bacteria during infections using an adaptable set of bioluminescent, fluorescent and colorigenic fusion vectors. *PLoS One* **6**: e20425.

Westra, E.R., Pul, Ü., Heidrich, N., Jore, M.M., Lundgren, M., Stratmann, T., et al. (2010) H-NS-mediated repression of CRISPR-based immunity in *Escherichia coli* K12 can be relieved by the transcription activator LeuO. *Mol. Microbiol.* **77**: 1380-1393.
